# Best practices on the differential expression analysis of multi-species RNA-seq

**DOI:** 10.1186/s13059-021-02337-8

**Published:** 2021-04-29

**Authors:** Matthew Chung, Vincent M. Bruno, David A. Rasko, Christina A. Cuomo, José F. Muñoz, Jonathan Livny, Amol C. Shetty, Anup Mahurkar, Julie C. Dunning Hotopp

**Affiliations:** 1 Institute for Genome Sciences, University of Maryland School of Medicine, Baltimore, MD 21201 USA; 2 Department of Microbiology and Immunology, University of Maryland School of Medicine, Baltimore, MD 21201 USA; 3 Infectious Disease and Microbiome Program, Broad Institute, Cambridge, MA 02142 USA; 4 Greenebaum Cancer Center, University of Maryland, Baltimore, MD 21201 USA

**Keywords:** RNA-Seq, Transcriptomics, Best practices, Differential gene expression

## Abstract

Advances in transcriptome sequencing allow for simultaneous interrogation of differentially expressed genes from multiple species originating from a single RNA sample, termed dual or multi-species transcriptomics. Compared to single-species differential expression analysis, the design of multi-species differential expression experiments must account for the relative abundances of each organism of interest within the sample, often requiring enrichment methods and yielding differences in total read counts across samples. The analysis of multi-species transcriptomics datasets requires modifications to the alignment, quantification, and downstream analysis steps compared to the single-species analysis pipelines. We describe best practices for multi-species transcriptomics and differential gene expression.

## Introduction

Transcriptomics experiments measure the underlying transcriptional signatures responsible for observed phenotypes [1–3]. By assessing mRNA profiles, it is possible to interrogate the specific genetic processes underlying, and giving rise to, specific phenotypes of interest. The use of transcriptomics has expanded to evaluate the transcriptional profile of other RNA populations [4], such as rRNAs [5], miRNAs [6–8], tRNAs [9, 10], and other small RNAs [11–14]. Traditional transcriptomics analyses usually identify transcriptional alterations in a single organism. However, biological processes often involve the interactions of multiple organisms, and interrogating the transcriptional profile of only one organism of a multi-organism system is insufficient to fully understand the biological system. This is especially important in the context of host-pathogen interactions, in which a holistic view of the biological system can aid in better understanding the system in ways to provide alteration, like the development of novel treatment therapeutics. But it is also important in the study of host-endosymbiont systems. To address this, researchers have developed methods to interrogate the transcriptome of multiple organisms from a single sample. Dual-species transcriptomics or dual-RNA-seq studies use transcriptomics to assess the transcriptional profiles of multiple organisms originating from the same sample [15].

The first dual-species transcriptomics studies were used to analyze interactions between the eukaryotic and prokaryotic organisms in host-pathogen systems [16–19]. However, compared to typical transcriptomics studies, dual-species transcriptomics studies are technically challenging due to a difference in the proportion of reads from the major and minor organisms in the system, where major and minor refer to transcript abundance. The organisms studied in dual-species transcriptomics experiments are present in different relative abundances and while the read proportions between the two organisms differ by system, most infection models, particularly biologically relevant models, have the total RNA content of the host vastly outnumbering microbe [15]. In the cases where the number of microbial cells are more limited, enrichment methods are needed to derive a meaningful number of reads from the minor organism for statistically robust analyses. As library enrichment methods have improved, dual-species transcriptomics studies expanded to include the study of eukaryote-eukaryote and prokaryote-prokaryote systems. As an example, dual-species transcriptomics has been used to study fungal interactions with numerous mammals and plants [20–25]. In prokaryote-prokaryote systems, dual-species transcriptomics has been used to study transcriptional profiles in prokaryotic biofilm interactions [26]. More recently, multi-species transcriptomics experiments have been conducted examining bacteria-eukaryote-eukaryote interactions in an endosymbiont-parasite-vector system [27]. With this increasing complexity, best practices are needed for properly designing and conducting a differential expression analysis within a multi-species transcriptomics experiment.

Here, we describe best practices for multi-species transcriptomics experiments from the initial experimental design to the downstream differential expression analysis, highlighting important considerations that should be taken for these multi-species analyses when compared to traditional single-species transcriptomics analyses. While we note currently available kits and tools for each step of the analysis, our goal here is neither to provide a comprehensive list of tools for each task nor to define the best tool. Similar to previous transcriptomics best practices studies [28, 29], our objective is to provide a guide for conducting a multi-species transcriptomics study from start to finish while highlighting considerations specific to multi-species transcriptomics studies.

## Sample preparation and sequencing

### Sample preparation

Sample preparation for multi-species RNA-Seq experiments requires maximizing the number of reads from the minor organism relative to the major organism. Even when the cells of the two organisms are present in equal numbers, the RNA molecules can differ in abundance by orders of magnitude. A single mammalian cell contains approximately two orders of magnitude more RNA than a single bacterial cell [15]. Enriching for mRNA from the minor organism has been reviewed previously [15, 30] and can be done using physical methods prior to sequencing, such as fluorescence-activated cell sorting [13, 23, 31, 32], laser capture microdissection [16], or differential lysis [33], all of which can also serve other purposes, like enriching for a select population of cells. Techniques with longer processing time need to incorporate steps to minimize further changes in the transcriptome during sample preparation including through the use of RNA stabilization reagents. Selection of the right reagents depends upon the system and techniques used (e.g., as described in [13] and recently reviewed by [30]). Enrichment for the minor organism can also be achieved by enriching for minor organism transcripts prior to sequencing using rRNA depletions [34] or custom RNA-Seq capture panels [35].

### Estimating the proportion of RNA from both the major and minor organisms

The design of a multi-species transcriptomics experiment is heavily influenced by the proportional composition of the organisms in the system of interest. It is important to define a target number of reads for each organism of interest and develop a sample preparation, enrichment, and sequencing strategy that can generate the target number of reads for the lowest cost and without introducing substantial bias. Thus, the relative proportions of each organism should be measured with techniques like qRT-PCR or limited test sequencing, which are then used to calculate a sufficient number of reads (described further below) to be sequenced to ensure adequate representation for all target organisms. For samples that include prokaryotes, methods for qRT-PCR and library construction should not rely solely on techniques that prime from the polyA-tail, since bacterial RNA transcripts largely lack polyA-tails. The optimal library size varies between different experiments and systems, and since fewer reads are needed when organisms have fewer transcripts, experimental designs must also consider the number of transcripts in the target organisms. Greater read depth is also needed if looking for rare transcripts and/or examining transcript isoforms, which may also require longer reads to fully resolve. CPM thresholds and saturation curves, both discussed in detail below, can be useful tools in assessing if a sample has been sequenced to sufficient depth for robust and rigorous differential expression analysis. While all of this can sometimes be achieved without enrichment and by sequencing for each organism separately, many experimental systems require the construction of multiple libraries using differential enrichment strategies.

### rRNA and polyA-RNA depletion and enrichment strategies for short-read sequencing

**Fig. 1:**
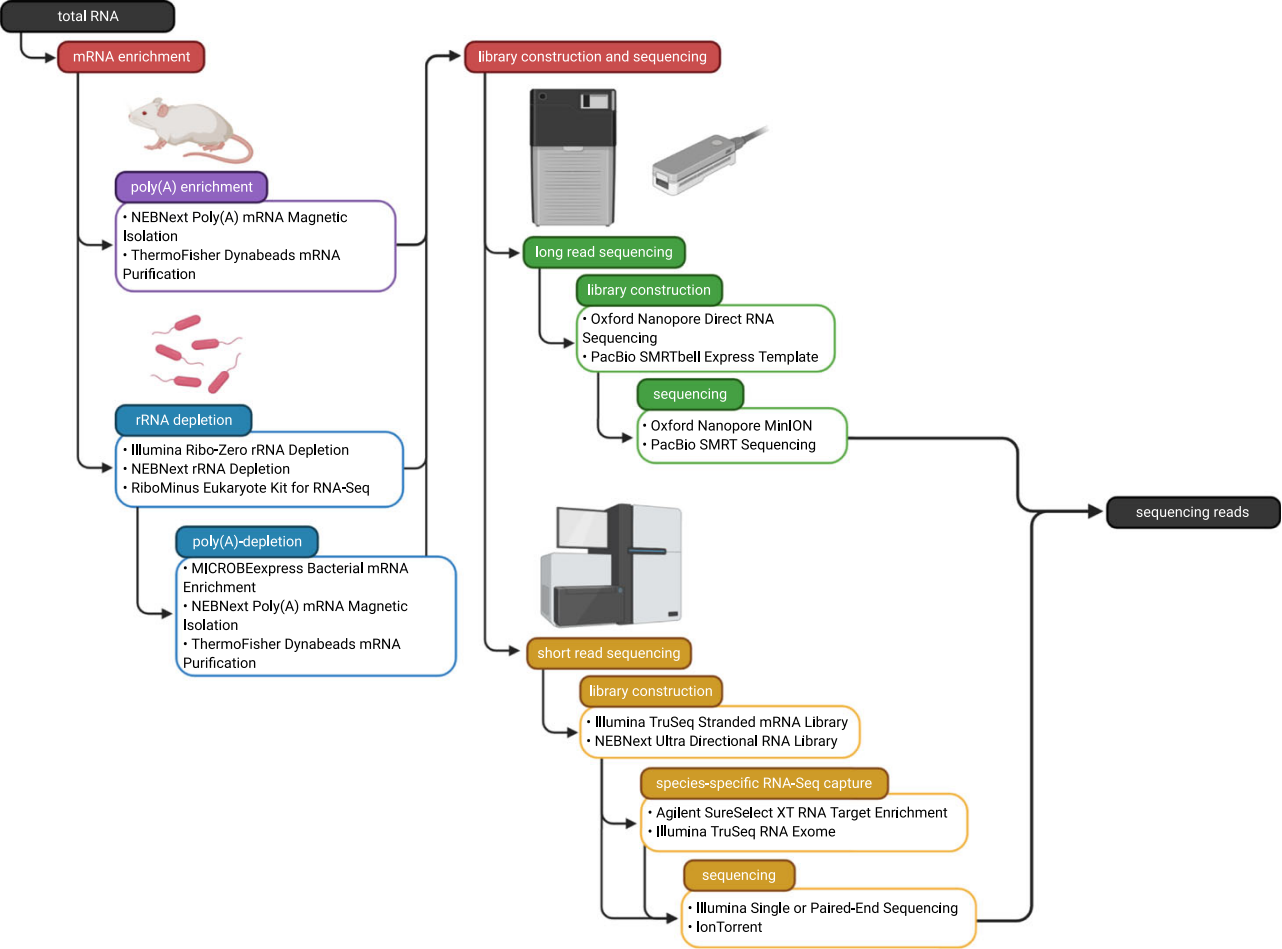
A general workflow for the enrichment, library preparation, and sequencing steps of a typical multi-species RNA-Seq analysis. Created with BioRender.com

When the major and minor organisms are all eukaryotes and the minor organism is at sufficient abundance, the transcriptomes of the major and minor organisms can be analyzed using libraries made following only polyA-enrichment. But when one of the organisms is a prokaryote, rRNA depletion is typically required with a kit that works on all organisms in the mixture. Total RNA is rRNA-depleted by selective removal using products such as the Illumina Ribo-Zero rRNA removal kit or the NEBNext rRNA depletion kit. In some cases, the difference in abundance of the major and minor member is too great requiring separate sequencing of the major and minor members. For obtaining the bacterial component of a bacteria-eukaryote sample, poly(A) depletion can be combined with the rRNA depletion, enriching for prokaryotic mRNA. Kits like the NEBNext Poly(A) mRNA Magnetic Isolation Module and the ThermoFisher Dynabeads mRNA Purification Kit are typically used for poly(A) enrichment with magnetic beads hybridized to oligo (dT) residues being used to extract polyadenylated transcripts (Fig. 1). Typically for poly(A) enrichments, after hybridization the supernatant is discarded and the poly(A)-selected RNA can be eluted from the beads. For poly(A) depletions, the supernatant is instead retained. Poly(A)- and rRNA-depleted RNA samples are enriched for all non-polyadenylated non-rRNA transcripts which include prokaryotic transcripts as well as eukaryotic RNAs that are not polyadenylated, like some long ncRNAs [36]. While effective in some systems, there are cases where sequencing after a rRNA-, poly(A) depletion is unable to yield a sufficient number of prokaryotic reads (e.g., obtaining sequencing data from the *Wolbachia* endosymbiont *w*Bm in *B. malayi* infected mosquitos [27]). In these instances, targeted transcriptomics capture approaches may be required to sufficiently enrich for reads originating from a specific organism [27, 37, 38].

### Targeted capture

For multi-species transcriptomics experiments involving eukaryote-eukaryote or prokaryote-prokaryote relationships, rRNA and polyA-RNA enrichments and depletions cannot be used to differentiate between the two organisms and enable enrichment of the minor organism, when needed. In these cases, targeted capture approaches, also referred to as CaptureSeq or Hybrid Capture, can be used to enrich for transcripts originating from an organism of interest [35, 37, 39, 40]. Targeted capture experiments rely on the use of probes designed to specifically hybridize to sequences in a target organism. With targeted capture methods, enrichments of up to 2242-fold fold have been reported with positive linear correlations (*r*^2^ = 0.56–0.87) relative to their counterparts that used rRNA and polyA depletions [40].

For example, one model of lymphatic filariasis includes three organisms: the vector host *Aedes aegypti*, the filarial nematode *Brugia malayi*, and the nematode’s bacterial *Wolbachia* endosymbiont, *w*Bm. At 18 h post-infection of *A. aegypti* with *B. malayi*, an enrichment of polyadenylated reads performed on total RNA yields 82.4% of reads mapping to *A. aegypti* and 0.4% of reads mapping to *B. malayi* [27, 40]. Using a targeted capture panel designed for *B. malayi*, a 146-fold enrichment of *B. malayi* reads can be obtained.

Targeted captures also provide an advantage when rRNA and polyA-RNA enrichment and depletion methods are unable to extract sufficient reads from the minor organism of the study, usually because the minor organism is of such low abundance. From the same mosquito/nematode/bacteria sample, rRNA and poly(A) depletion performed on total RNA yielded 122 (< 0.1%) of reads mapping to the bacteria, *w*Bm. Using a targeted capture designed for *w*Bm on total RNA, the number of *w*Bm mapped reads increased to 703,956 (0.9%) [40], which is on average over 850 reads/gene.

Targeted capture relies on knowing the transcript sequences. Therefore, transcript sequences must already be known or inferred through either transcriptome sequencing/assembly or whole genome sequencing/assembly/annotation. Since oligonucleotides are custom designed and synthesized for each transcript, the method can be expensive. A method for capturing after library multiplexing could help drive down cost. Lastly, the results are biased by the oligonucleotides in the capture. For instance, leaving out the rRNA genes is desirable as it limits the presence of rRNA following capture. But any other region not included will also not be captured. This limits the potential to discover new transcripts, or analyze newly discovered transcripts, unless oligonucleotides are used that tile both strands of the whole genome.

### Enrichment methods for long-read sequencing

The larger scale of sequence reads obtained by short-read sequencing technologies relative to their long-read counterparts provides higher power that is indispensable for differential expression analyses (Fig. 1). Additionally, for low abundance minor organisms in multi-species transcriptomics experiments, the greater number of reads conferred by short paired-read sequencing is advantageous with respect to detection of the minor organism. However, when a high-quality reference is not available for mapping and a de novo transcriptome assembly is required, long-read sequencing may be advantageous to improve assembly quality and detect isoforms. Long reads may also have advantages in the accurate identification of transcript isoforms [41, 42]. However, the relative abundance of the major and minor organism must again be considered.

While transcript variants have been less studied in bacteria, there is increasing evidence of alternate transcription start sties and termination sites internal to operons, as well as antisense transcription, cis-regulatory elements, and riboswitches in 5′-untranslated regions [43–45]. Therefore, methods to obtain long bacterial reads are needed, particularly ones that can be leveraged for multi-species RNA-seq analyses. Long-read SMRT sequencing can be supplemented with a Pacific Biosciences IsoSeq protocol that includes a poly(A) enrichment and rRNA depletion step [46] while Oxford Nanopore Technologies direct RNA sequencing can be performed on either in vivo polyadenylated mRNA from eukaryotes or in vitro polyadenylated RNA from any organism, including bacteria [47]. SMRT-Cappable-seq can also be used to enrich for bacterial RNA, generating long bacterial IsoSeq reads using a protocol that adds a desthio-biotinylated cap to 5′-triphosphorylated primary prokaryotic mRNA transcripts followed by polyadenylation [48]. The biotinylated cap can then be enriched and sequenced, enabling the identification of the 5′-end, operons, and transcript variants. SMRT-Cappable-seq RNA can also be sequenced using the Oxford Nanopore technology to generate similar data by directly sequencing RNA, allowing for the additional potential analysis of RNA modifications. However, the reactions require a large amount of RNA that may be difficult to obtain in many systems, particularly when interrogating a low abundance minor member. Methods for targeted enrichment of cDNA constructed from long transcripts have been reported [49, 50] that could be used for cDNA-based IsoSeq or ONT libraries, but do not scale to obtaining whole transcriptome enrichment of the complete transcriptome of the minor organism without having all ORFs cloned in the minor organism. Methods designed to capture long DNA fragments will likely capture from cDNA, but methods are needed for long RNA capture for direct sequencing with ONT. As mentioned with capture of Illumina libraries, these methods require a priori knowledge of the genome or transcriptome to design the baits and are limited by the bait design.

## Alignment and quantification

### Differences in the analysis of prokaryotic and eukaryotic RNA-Seq data

There are important differences in analyses between eukaryote and prokaryote data. Due to splicing observed in eukaryote transcripts, separate aligners and options are frequently used for prokaryotes and eukaryotes. The human and mouse genomes represent two of the best annotated genomes to date, such that the sequences of entire transcripts are known. In comparison, genomes of non-model organisms have less established gene models, often containing only coding sequences and lacking UTR sequences. While de novo transcriptome assemblies, using tools such as Cufflinks [51], Oases [52], rnaSPAdes [53], Trans-ABySS [54], or Trinity [55], could serve as a potential solution, it is often difficult to perform on the minor organisms in multi-species transcriptomics experiments due to a lack of adequate sequencing depth. While sequencing pure cultures of an organism in different conditions can provide good coverage of a transcriptome for a de novo assembly, such methods are not as straightforward for obligately host-associated organisms.

### Quality control and read alignments

**Fig. 2:**
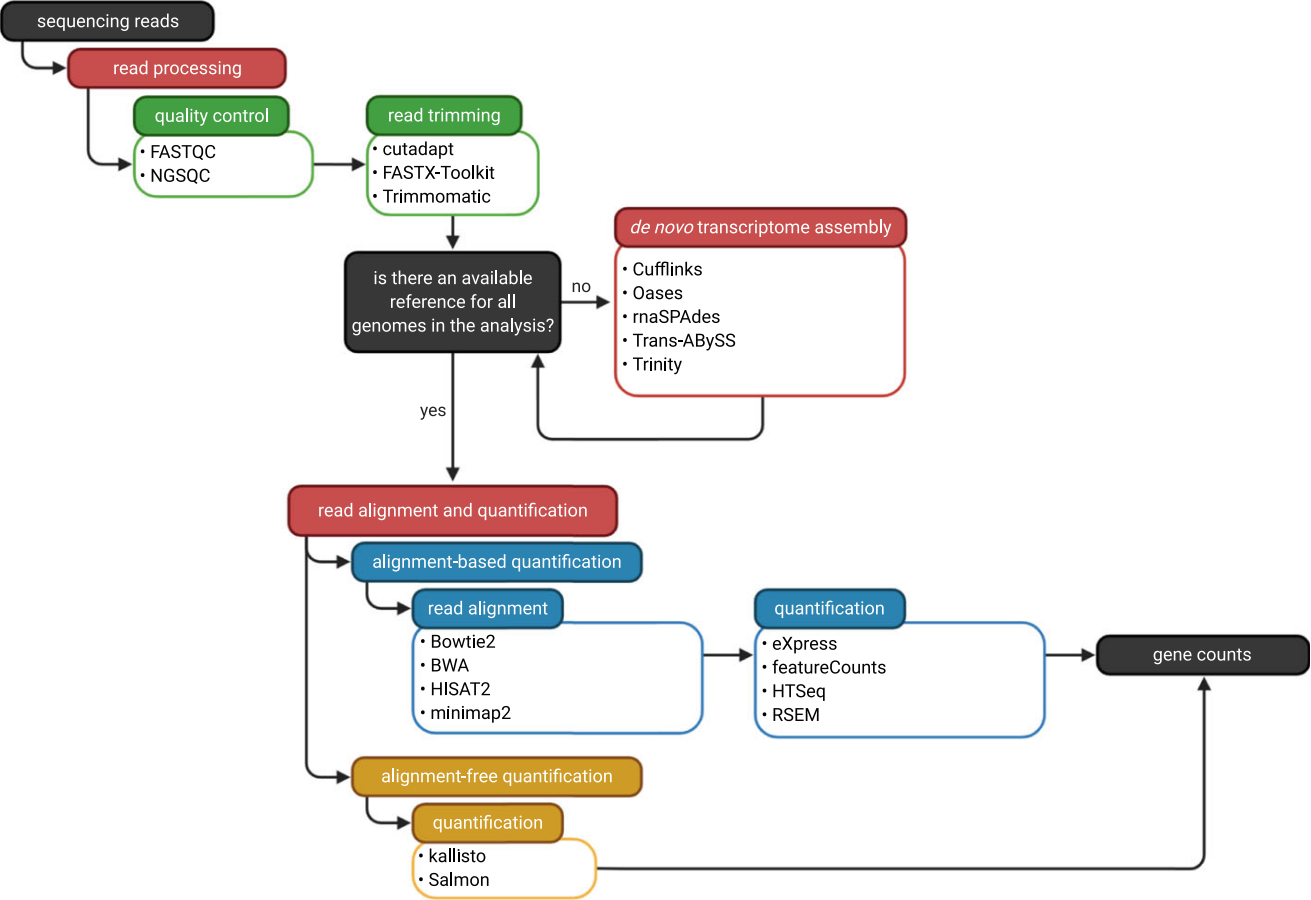
A general workflow for the read processing, alignment, and quantification steps of a typical multi-species RNA-Seq analysis. Created with BioRender.com

Following quality control of the sequenced reads and their subsequent trimming, reads are typically aligned to a reference genome or transcriptome for each species or to a composite multi-species reference (Fig. 2), although alignment-independent methods can also be used. Raw read data should be quality-controlled with tools, such as FASTX-toolkit [56], FASTQC [57] or NGS QC [58], to examine the GC content, base quality score, and the total number of reads sequenced. Reads should be trimmed with Cutadapt [59] or Trimmomatic [60]. For prokaryotes, splice-agnostic aligners such as Bowtie [61] or BWA [62] can be used, while for eukaryotes, splice-aware aligners such as HISAT2 [63] or STAR [64] are frequently used. Aligner issues have been identified because aligners are primarily tested on simulated data and typically human data [65], illustrating that more testing with real data from diverse organisms would be beneficial. Because most aligners have been designed with the intent of aligning reads to a single organism, most tools are not optimized for multi-species data sets. Using a combined reference containing the nucleotide genome or coding sequences of all target organisms in a multi-species transcriptomics analysis mitigates the number of incorrect mappings and should be used in most cases [66, 67] and should be splice-aware if any organism has splicing. As an additional precaution, reads with equal mapping to two (or more) organisms, expected to be few, can be removed from the analysis [23], with the exception of endosymbiont/host systems with extensive lateral gene transfer, like *Wolbachia* endosymbionts and their hosts [68].

The initial seed length is also important for the correct mapping of reads. When applied to multi-species datasets, the aligner BWA-MEM has been found to sometimes yield a substantial number of reads mapping to the incorrect organism when run using the default seed length of 19 nt and a single reference [66]. This misalignment can be avoided when references are merged, but when they cannot be merged (e.g., because the aggregate genome size of the organisms is too large for the aligner), increasing the seed length can improve the analysis [66].

### Alignment-dependent transcript quantification

Alignment files obtained from read mapping are inputs for transcript quantification using tools such as featureCounts [69] or HTSeq [70] (Fig. 2). Using reference annotation in a GFF/GTF file, alignment-dependent quantification tools take the coordinates of mapped reads and counts fragments per gene based on the overlap between the mapping coordinates of the read and any specified feature in the annotation file. The tools featureCounts and HTSeq use genome mapping with annotation while other quantification tools such as eXpress [71] and RSEM [72] require mapping to transcriptome sequences. The tools eXpress and RSEM use on-line or batch expectation maximization (EM) algorithms, respectively, to assign ambiguous fragments to target sequences based on probability [71, 73]. Additionally, the quantification tool RSEM can perform the read alignment step prior to quantification or directly use an alignment file as an input [72]. While a good option where the set of transcripts has been previously well defined, de novo transcriptome assemblies can be difficult, nuanced, and imprecise, making extensions even with weak support [74, 75], which frequently preclude the use of transcriptome-alignment-based tools. Mapping to complete genome sequences provides greater specificity in read mapping, particularly for reads from unannotated transcripts [65] or unannotated portions of transcripts like 5′- and 3′-UTRs in genomes where only CDSs are annotated.

For genomes that have not been extensively characterized, transcript quantification is often performed at a coding sequence level rather than a transcript level. Yet, the widespread presence of operons in prokaryotes [76, 77] causes potential downstream issues [78]. Transcripts from operons contain multiple coding sequences in a single mRNA transcript, and the presence of operons can lead to very long transcripts in bacteria. The close proximity of genes in an operon leads to issues when attempting to quantify coding sequences rather than transcripts, leading to an underestimation in the read counts for genes encoded in operons, with smaller operonic genes being more heavily impacted [78]. FADU is prokaryote-specific read counting algorithm with an implemented EM algorithm [78]. FADU balances errors in bacterial read counting that was tested on simulated data and real data from multiple organisms [78], but more research and algorithm development are needed in this area.

### Alignment-independent transcript quantification

Alignment-independent tools quantify reads based on a pseudoalignment or quasi-mapping of read k-mers allowing for considerably faster compute times relative to alignment-dependent tools while obtaining similar results. As inputs, k-mer-based tools require an index generated from a nucleotide FASTA file containing the transcript sequences of the target organism along with paired-end FASTQ files. Alignment-independent tools available for transcript quantification include kallisto [79], Sailfish [80], and Salmon [81] (Fig. 2). References for alignment-independent approaches should include predicted transcript sequences from all organisms of interest to maximize the accuracy of the quantification tool. Similar to transcriptome-alignment-based quantification methods, the lack of high-quality transcriptome assemblies and/or transcript annotation may preclude the use of these tools. Recently, some issues have been identified with these tools as testing has heavily relied on simulated data and reads from unannotated transcripts can be misassigned, although suggested improvements and implementation of an option in Salmon may alleviate some of these issues [65]. However, testing is needed on more diverse data, particularly prokaryotes since transcript structural variation is quite different and intron/exon differences cannot be used to disentangle read counts.

## Downstream analyses

### Saturation curves

**Fig. 3:**
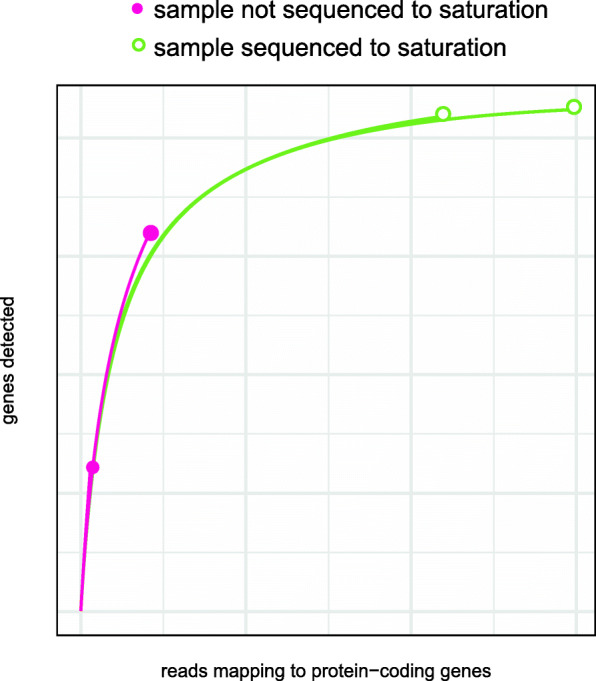
Examples of saturation curves for two samples that reach saturation and two samples that do not reach saturation

To determine if a sample has been sufficiently sequenced, a saturation curve can be generated with software like vegan in R [82]. To generate saturation curves, subsets of the reads in a sample are taken and the number of detected genes in each of these subsets is plotted [83] (Fig. 3). In other words, the number of reads in a subset are plotted on the *X*-axis and the number of transcripts detected with that subset are plotted on the *Y*-axis with the final value in the plot being the number of transcripts detected in the complete dataset. A sample sequenced to saturation plateaus, such that saturation is assessed by looking at the shape of the curve. It is important that the emphasis is not on the height of the plateau since samples can have different subsets of genes transcribed such that they plateau at different levels. For example, in an analysis of the life cycle of a filarial nematode, male samples consistently plateau above other samples, likely because of the large number of male-specific transcripts only in these samples [27]. In the case that the curve is not reaching saturation, it likely indicates that the sample has not been sequenced to a sufficient depth. In these cases, the library could be queued for additional sequencing and fastq files merged from multiple runs. When further sequencing is not possible, samples might be removed from the experiment [27], new libraries constructed with enrichment [27], or the analysis could be adjusted accordingly, (e.g., examining the rank abundance of the most highly expressed genes can form the basis for new testable hypotheses [25]). Analysis methods presented below in addressing batch effects may be beneficial. An alternative or compliment to saturation plots involves plotting the number of new genes added with each subsequent addition of a subset of reads in a saturation analysis; such a plot shows a decay that also plateaus indicating the limits of detecting new genes [83].

### Expression threshold

Genes with low abundance or unusually high abundance may need further examination before downstream analyses. For low abundance genes, a minimum expression depth threshold is needed that is applied evenly to each organism in all samples. The differential expression tools DESeq2 [84] and edgeR [85] both employ thresholds to ensure that genes have sufficient reads to be considered for downstream analyses. By default, DESeq2 uses a prefiltering step that excludes all genes with < 10 reads and a later filtering step on the mean of normalized counts [84]. The edgeR manual suggests using a counts-per-million (CPM) threshold to establish the minimum number of reads that a gene must have to be considered valid [85], and the CPM threshold is implemented to ensure that it is applied equally across samples regardless of sequencing depth differences between samples.

Examining extremely high abundance genes may also need to be addressed, although the TMM algorithm in EdgeR was designed for these issues [86]. Erroneously predicted CDSs within rRNA genes can have very high counts, which can be remedied by examining and correcting the annotation. High counts have also been an issue in *Wolbachia* transcriptomes for the 6S RNA, a noncoding RNA and global transcriptional regulator that associates with RNA polymerase [87]. The *Wolbachia* 6S RNA is differentially expressed and possibly associated with control of intracellular replication and growth [88, 89]. In the 2005 *Wolbachia* strain wBm annotation, the 6S RNA was not annotated and an adjacent CDS (Wbm0439) had a misannotated start site placed within the 6S RNA [90]. This misannotation combined with the high levels of 6S RNA expression (> 75% of the non-RNA in some samples [27]) impeded analysis of differentially expressed genes with WGCNA. This was remedied by using updated NCBI annotation, limiting the analysis to CDSs, and including a separate analysis of the 6S RNA [27]. Heat maps of TPM values can be helpful in identifying these issues.

### Clustering to identify technical artifacts

**Fig. 4:**
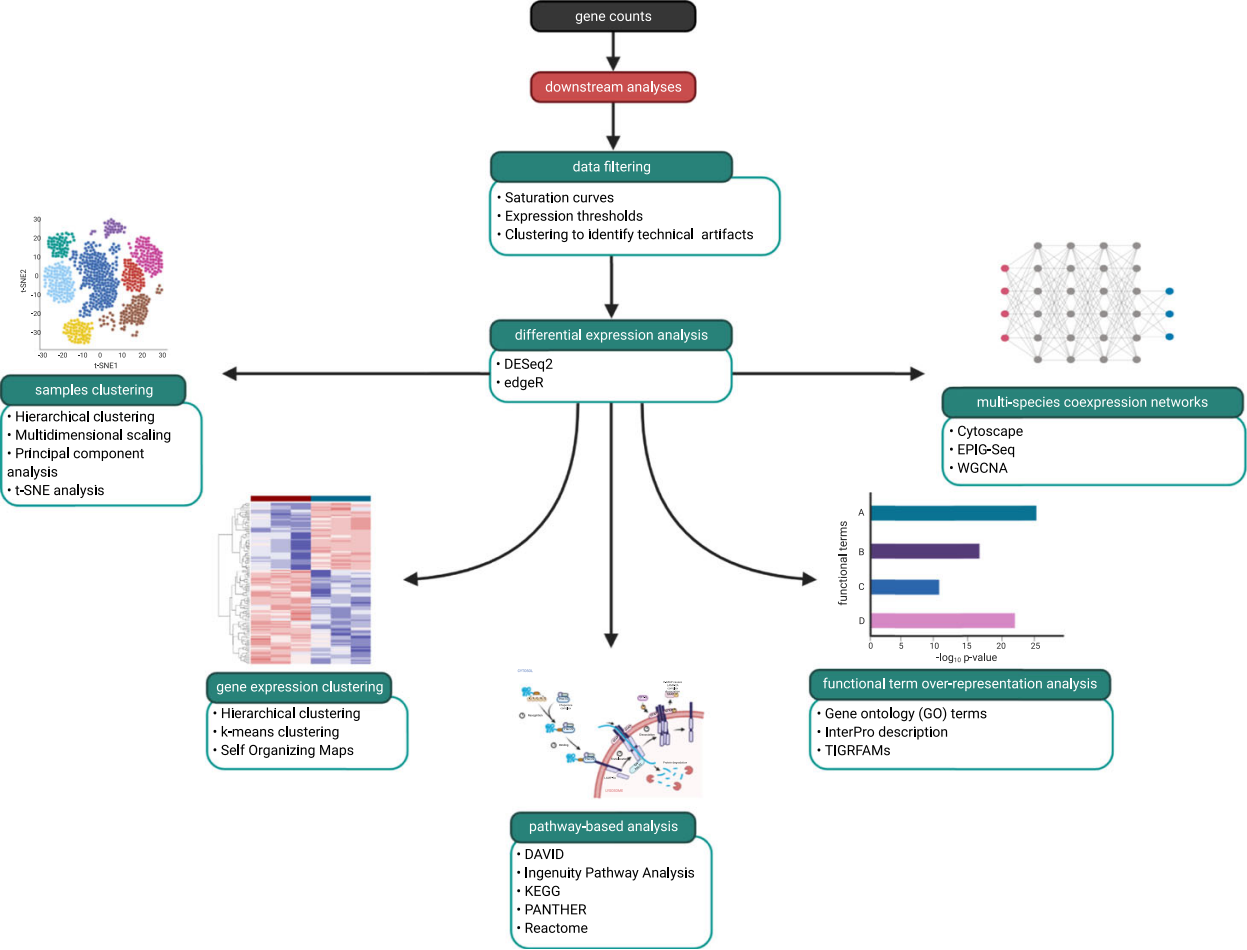
A general workflow showing examples of downstream analyses for a typical multi-species RNA-Seq analysis. Created with BioRender.com

Before other downstream analyses, clustering analyses such as principal component analyses (PCA) or hierarchical clustering should be completed to ensure that samples cluster together based on experimental design, such as biological replicates, and not by technical factors, like the number of reads sequenced, library preparation, or sequencing runs (Fig. 4). If technical artifacts are suspected, an interrogation of batch effects as described further below is warranted. The R package WGCNA has functions to determine outlier samples using hierarchical clustering of the samples using gene expression values [91]. Statistical support should be used to measure confidence in the generated clusters, such as with bootstrap supports in the case of hierarchical clustering. In the case that biological replicates do not cluster with one another, the samples must be examined further for potential sequencing artifacts or inadequate sequencing depth. If a small number of samples display aberrant clustering, such as due to low sequencing depth, removing them may be a preferred option where sufficient samples remain for the planned analyses. Clustering should be performed for the data for each organism in the samples separately, as the transcriptional variation patterns can differ between organisms in the magnitude and direction of change.

For the comparison of transcript abundance across multiple samples, transcript counts need to be normalized in each organism by individual transcript lengths and total library size. Due to the differences in transcript abundances between the major and minor organisms, counts for each organism should be normalized independently using transcript per million (TPM) calculations [73]. TPM values are calculated by dividing all read counts by the length of each gene in kilobases to obtain a reads per kilobase (RPK) value for each gene [92]. The RPK value for each gene is then divided by the sum of RPK values divided by 1,000,000. While RPKM and FPKM calculations are also used for normalization, the sum of the RPKM and FPKM values differ between samples with differing numbers of reads, which can result in disproportionate comparisons [92].

### Batch effects

Frequently, experimental designs assume that the variation between treatments is larger than biological or technical variation between samples within a treatment, but there are situations where this is not true. This biological or technical variation (referred to as batch effects) leads to unwanted data variation and other normalization methods can be used to remove these systematic artifacts. RUV normalization uses replicate libraries or spike-in controls [93], or alternatively negative control genes or samples [94], to normalize for batch effects. The tool limma addresses this issue by modeling heterogeneity at the sample and observational levels, yielding fewer false discoveries [95]. SVA [96] and PEER [97] are two additional methods for detecting batch effects in transcriptomics data that rely on different statistical methods and can be applied when needed in an analysis.

### Differential expression analysis

In the case of multi-species transcriptomics analyses, separate differential expression analyses are typically conducted for each organism. DESeq2 [84] and edgeR [85] both use raw read counts instead of normalized counts to perform differential expression analyses (Fig. 4), as reviewed previously [98]. Prior to conducting differential expression analyses, both tools have internal methods to normalize across different library sizes along with the difference in the relative size of the target transcript, with DESeq2 using a relative log expression (RLE) normalization [99] and edgeR using a trimmed mean of m-value (TMM) normalization [86]. Both methods along with a third method, median ratio of normalization (MRN) [100], yield similar results when used for the pre-processing steps of a differential expression analysis [98]. For differential expression analyses with kallisto or Salmon, the tools sleuth [101] and Swish [102] account for the uncertainty in the alignment-free quantification, reducing technical biases or inferential variance.

Prokaryotic differential expression analyses are typically performed at the coding sequence (CDS) level, despite operons being widespread in bacterial genomes, with 630–700 operons being estimated in the *Escherichia coli* genome [103]. However, differential expression is more accurate when applied to transcripts as opposed to CDS. To illustrate this, we used an existing simulation ([78]; https://github.com/IGS/FADU) of an RNA-Seq experiment for *E. coli* K-12 substrain MG1655 using Polyester [104] to model three hundred 2-fold over- and under-expressed transcripts using transcript models obtained from OperonDB [105] with 582 overlapping transcripts. The simulation had 592,066–625,563 reads per sample and consisted of two conditions of two replicates each. A total of 556 of the 1973 *E. coli* transcripts (1246 of the 4419 genes) were simulated as differentially expressed with the remainder being not differentially expressed. We assessed the performance of 13 quantification methods, using the tools FADU [78], featureCounts [69], HTSeq [70], kallisto [79], and Salmon [81], paired with two differential expression tools, DESeq2 [84] and edgeR [85], using either transcript or gene models (Table 1). Across all methods of quantification and differential expression, we see marked improvement in the accuracy of detecting differential expression when using transcript models with an increase in detection of differentially expressed genes of 21.9–40.4% for DESeq2 and 1.8–20.1% for edgeR (Table 1). This suggest that many differentially expressed genes are currently being overlooked. The number of transcripts being falsely deemed as differentially expressed (false positives) was more constant when using genes with the exception of the counting algorithms featureCounts -O and HTSeq --nonunique all, which assign full counts to all transcripts that a given read pair overlaps. This suggests that for most counting algorithms using prokaryotic transcript annotations allow for substantially more accurate prediction of differentially expressed genes.

**Table 1:** Differential expression analysis comparing gene vs. transcript models using simulated data

Quantification method	DESeq2 DE genes detected	DESeq2 DE transcripts detected	DESeq2 false positive DE genes	DESeq2 false positive DE transcripts	edgeR DE genes detected	edgeR DE transcripts detected	edgeR false positive DE genes	edgeR false positive DE transcripts
FADU	684 (54.9%)	440 (79.1%)	7 (0.56%)	11 (2.0%)	947 (76.0%)	439 (79.0%)	34 (2.7%)	12 (2.2%)
FADU -em_iterations 10	686 (55.1%)	438 (78.8%)	10 (0.8%)	12 (2.2%)	955 (76.7%)	436 (78.4%)	37 (3.0%)	12 (2.2%)
FADU -remove_multimapped	702 (56.3%)	435 (78.2%)	4 (0.32%)	5 (0.9%)	974 (78.2%)	447 (80.4%)	32 (2.6%)	9 (1.6%)
featureCounts	694 (55.7%)	434 (78.1%)	5 (0.4%)	5 (0.9%)	936 (75.1%)	441 (79.3%)	24 (1.9%)	10 (1.8%)
featureCounts -O	775 (62.2%)	515 (92.6%)	18 (1.44%)	54 (9.7%)	1008 (80.9%)	522 (93.9%)	43 (3.5%)	61 (11.0%)
featureCounts -O -fraction	734 (58.9%)	505 (90.8%)	14 (1.12%)	20 (3.6%)	1000 (80.3%)	528 (95.0%)	47 (3.8%)	44 (7.9%)
HTSeq -m union	644 (51.7%)	428 (77.0%)	4 (0.32%)	5 (0.9%)	909 (73.0%)	436 (78.4%)	40 (3.2%)	12 (2.2%)
HTSeq -m intersection-strict	607 (48.7%)	436 (78.4%)	1 (0.08%)	5 (0.9%)	803 (64.5%)	442 (79.5%)	24 (1.9%)	14 (2.5%)
HTSeq -m intersection-nonempty	656 (52.7%)	436 (78.4%)	3 (0.24%)	5 (0.9%)	903 (72.5%)	442 (79.5%)	31 (2.5%)	14 (2.5%)
HTSeq -m union -nonunique all	769 (61.7%)	509 (91.6%)	18 (1.44%)	48 (8.6%)	1005 (80.7%)	519 (93.4%)	47 (3.8%)	65 (11.7%)
kallisto	675 (54.2%)	526 (94.6%)	9 (0.72%)	11 (2.0%)	946 (75.9%)	532 (95.7%)	41 (3.3%)	22 (4.0%)
Salmon -validateMappings	676 (54.3%)	525 (94.4%)	4 (0.32%)	8 (1.4%)	946 (75.9%)	534 (96.0%)	44 (3.5%)	25 (4.5%)
Salmon -validateMappings -allowDovetail	675 (54.2%)	525 (94.4%)	4 (0.32%)	9 (1.6%)	946 (75.9%)	534 (96.0%)	46 (3.7%)	23 (4.1%)

Despite the substantial improvement that a transcript annotation confers to a differential expression analysis, most prokaryotic differential expression analyses are limited to being conducted at the gene level. Because of the difficulty in annotating full-length transcripts for non-model organisms, particularly those with polycistronic transcripts and/or a high coding density, there is currently no easy solution to this problem in prokaryotic differential expression analyses [106]. This is another area for further research and algorithm development, perhaps incorporating long reads to annotate polycistronic transcripts.

### Clustering genes by expression pattern

Methods like hierarchical clustering, k-means clustering, and self-organizing maps can be used to identify clusters of coordinately regulated genes with similar expression patterns [107, 108] (Fig. 4). The representative expression pattern for each of these clusters can be identified by taking the average of the *z*-score of the log-transformed expression values for each of the sample. The *z*-score is the number of standard deviations that a value for a given gene in a given sample is away from the mean of all the values for all the samples for the same gene. A *z*-score of -2 means that this value is 2 standard deviations lower than the mean across all the samples. It is an effective tool for normalizing prior to visualization particularly when there is not a clear reference sample. When a reference sample is available that all samples are compared to, the log-fold change can be shown relative to the reference. Clustering on counts or log-transformed counts can also be useful and is often the most intuitive. For studies with multiple sample types, like time courses, algorithms like WGCNA can be helpful to identify co-regulated genes. WGCNA constructs a co-expression network using normalized expression values to form co-expression clusters that contain the genes regulated to the same extent under the same conditions, although sometimes in opposite orientations [91]. Hierarchical clustering and dynamic tree cutting of the co-expression network allows for the identification of distinct expression clusters. From WGCNA, each expression cluster has an eigengene that indicates the major representative expression pattern of the contained genes, while the inverse of the eigengene can be used to identify genes with the inverse pattern of expression. Similarly, EPIG-Seq [109] is another clustering tool that extracts gene profiles from count data and uses them to create clusters of genes based on their expression profiles. EPIG-Seq then assesses the significance of the clustering of co-expressed genes to their respective patterns and assigns a *p* value to each gene.

When there is a large difference in the number of genes between major and minor organisms, the normalized expression values for each of the target organisms may need to be clustered separately. Otherwise, the data from the major organism may seed almost all the recovered co-expression clusters such that only expression patterns from the major organism are recovered. Recovering expression modules separately for each organism in the study allows for the recovery of prominent co-expression patterns in all organisms of interest and those parallel patterns can be compared post hoc. Co-expression examined using WGCNA revealed the interplay of pathways between a *Wolbachia* endosymbiont and its *B. malayi* host over the life cycle of the parasite [110]. Clusters of HeLa/human genes were identified that have expression that correlates with a *Salmonella* gene using a *z*-score on counts that were analyzed with a Pearson correlation and *p* values [13]. In a study of *Haemophilus ducreyi* infection in humans, a generalized linear model was used to identify 106 bipartite networks containing 146 host genes and 114 bacterial genes [111].

### Gene co-expression network construction

Using tools such as WGCNA and EPIG-Seq, correlation matrices can be constructed for a set of differentially expressed genes across a multi-organism dataset (Fig. 4). These correlation matrices can be used to construct multi-organism expression networks that can be visualized with tools such as Cytoscape [112] in order to identify genes whose expression patterns are highly similar to one another. Additionally, the protein-protein interaction database STRING [113] can accept a list of differentially expressed genes for over 5000 organisms to construct protein-protein interaction networks. Using these network construction tools, differentially expressed genes can be narrowed down into smaller gene subsets for functional term enrichment or pathway-based analyses [114]. Construction of a genome-informed network representation of a parasite’s metabolic capabilities and integration of transcriptome data for both the nematode parasite *B. malayi* and its *Wolbachia* endosymbiont led to the identification of stage-specific metabolic dependencies and potential therapeutic targets, three of which were experimentally verified with human drugs [115].

### Functional analyses for sets of differentially expressed genes

The list of genes derived from differential expression and clustering tools are used to extrapolate biological significance from the input samples (Fig. 4). Using the functional annotations assigned to different genes, such as Gene Ontology (GO) terms [116, 117], InterPro descriptions [118], KEGG orthology IDs [119–121], or TIGRFAMs [122], differentially expressed genes or expression modules can be statistically analyzed for the over- or under-representation of specific functional terms. These lists of genes can also be used for gene set enrichment analyses using tools such as DAVID [123, 124]. By identifying significantly over- or under-represented functional terms, biological systems can be summarized into the general up- or downregulation of the broader functional processes in each of the different organisms in the multi-species system. Additionally, pathway enrichment analyses can be conducted using Ingenuity Pathway Analysis (IPA) [125] for pathway analysis from human gene lists, while Reactome and PANTHER [126] can be used for the pathway analysis of eukaryotic and prokaryotic organisms, further defining the metabolic pathways integral to a multi-species system. For example, use of IPA on the host transcriptional response to two different fungi led to identification of EGF receptor (EGFR) as a novel host target [24, 127]. Inhibition of EGFR signaling with cetuximab or gefitinib, which are both FDA-approved inhibitors of EGFR, leads to a reduction in invasion and damage during fungal infection, and gefitinib prolonged survival in a mouse model [127]. Unfortunately, IPA is limited to examining only human, mouse, or rat hosts.

## Other applications

### Single-cell multi-species transcriptomics

Developed in 2009, single-cell approaches to transcriptomics (scRNA-Seq) have been increasingly used to examine the transcriptome of individual cells compared to the population-level transcriptome of a sample [128–131]. By using individual cell isolation methods such as fluorescent activated cell sorting [23, 132, 133], laser microdissection [16], or micromanipulation methods [134–136], it becomes possible to isolate individual cells of interest, extract their RNA, and sequence their transcriptome individually. Additionally, there are entire suites of tools developed for scRNA-Seq analyses (for reviews, see [137, 138]).

The continued development of scRNA-Seq provides an additional tool for multi-species transcriptomics analyses. While traditional multi-species transcriptomics studies must factor the possibility of the transcript abundances of the major organism overwhelming the transcript abundances of the minor organism, single-cell isolation approaches allow for different cells belonging to the different organisms to be separated. Additionally, different populations of cells from the target organisms can be extracted and analyzed. This allows for separate bulk transcriptomes to be extracted from individual cells within a population and can reveal that the average transcriptional signal from a population may correspond to heterogenous gene expression. For example, infected and uninfected hosts cells can be distinguished. For both eukaryotic and prokaryotic microbes, the gene expression in different niches can be assessed including distinguishing intracellular or extracellular interactions with respect to the host. With intracellular bacteria, there may be both specific opportunities and challenges that relate to cell lysis and whether host and bacterial cells lyse under similar or different conditions. Some studies have carried out simultaneous analysis of single microbial and host cells, such as bacteria or fungi engulfed by macrophages [23, 31, 132, 133]. Due to the low input RNA of a single cell, the quality filtering and analysis methods vary from standard methods for bulk samples. Examining transcriptomic variation between multiple organisms in parallel can reveal profiles corresponding to states of each organism, as well as trajectories between stages, resulting, for example, in different infection outcomes [23].

As of now, prokaryotic scRNA-Seq approaches are lacking in that prokaryotes have extremely low RNA abundance, lack mRNA polyadenylation, and have thick cell walls [139]. While the recent development of techniques such as PETRI-seq [139] and microSPLiT [140] has enabled the capture of single-cell prokaryotic transcriptomes, increased sensitivity is likely needed before being able to properly interrogate the minor organism of multi-species host-pathogen systems. Additionally, no techniques have been demonstrated to be simultaneously applicable to eukaryotes and prokaryotes, limiting the use of scRNA-Seq in many host-pathogen systems. Despite this, further advancements in scRNA-Seq has the potential to allow for the interrogation of multi-species systems in greater detail than ever before.

### Metatranscriptomics

While multi-species RNA-Seq studies look at the interplay between a handful of defined organisms, metatranscriptomics studies are frequently used to characterize gene expression of all members within a given biological system. The design of metatranscriptomics studies requires further considerations in that a proper study design must account for the high diversity and complexity of the biological community of interest, which includes the relative ratios of its different members and the large dynamic range of transcript expression [141].

There are many tools for metatranscriptomics analyses (for review, [141]).The upstream pre-processing steps of a metatranscriptomics studies are similar to that of a dual-RNA-Seq study in that the same trimming and depletion tools can be used. For the metatranscriptomic analysis of the microbiome, mycobiome, or virome of a host-pathogen system [142], host reads must first be depleted to minimize the potential of host reads being misclassified as reads belonging to another taxa. This can be done through a subtractive alignment, by first mapping reads to the host genome and excluding all mapped reads [143], or using tools that perform a similar function, such as SortMeRNA [144]. Metatranscriptomic reads can then be taxonomically classified using tools such as Kraken2 [145], MetaPhlAn [146], or Centrifuge [147] to identify the transcriptionally active members of a community and functionally categorized using tools and pipelines such as FMAP [148], HUMAnN3 [149], or MetaTrans [150]. Differential abundance analyses of functional active taxa or transcripts can be identified using traditional RNA-Seq differential expression tools such as DESeq2 [84], edgeR [85], or limma [151]. Additionally, LEfSe [152] has been developed specifically for the identification of discriminating features between different groupings of samples in metagenome analyses.

The current limitations of metatranscriptomics lies in the limited reference databases available for both taxonomic and functional categorizations, leading to a large proportion of unclassified reads in the analysis. While this can be addressed through the use of tools for de novo metatranscriptomics assembly, such as IDBA-UD [153] and rnaSPAdes [53, 154], the results can be confounded due to repetitive patterns in different genes along with large variances in mRNA abundances due to both differences in expression and the abundances of different species [53, 154].

## Future directions

Transcriptomic approaches designed to study a single species in isolation are often inadequate for effectively profiling the transcriptomes of multiple species in the same sample. By enabling the simultaneous interrogation of gene expression in multiple organisms, multi-species transcriptomics provides key insights in the transcriptional networks and regulatory pathways that govern multi-organism interactions within complex biological systems. As enrichment methods, sequencing technologies, and analysis tools continue to develop, multi-species transcriptomics will yield more comprehensive and accurate maps of interactions between an increasing number and diversity of organisms. Importantly, multi-species transcriptomics will become an increasingly powerful tool to explore the interactions of microbial pathogens and their mammalian hosts, providing the foundation for novel therapeutic strategies that target as-of-yet unknown virulence factors and host defense pathways that would have remained hidden in traditional single-species transcriptomic analyses.

## Supplementary Information

**Additional file 1.** Review history.
