# Supplementary material for: Best practices on the differential expression analysis of multi-species RNA-seq
Source: Genome Biol. 2021 Apr 29;22:121. doi: 10.1186/s13059-021-02337-8 (PMC8082843; doi:10.1186/s13059-021-02337-8)
Supplement: Supplementary file 1 — Additional file 1. Review history. [file 13059_2021_2337_MOESM1_ESM.docx]

Review History

**First round of review**

**Reviewer 1**

**Comments to author:**

Chung et at. present a guidelines paper for RNA-seq analysis in multi-species experiments, i.e. with the RNA from more than one species is analyzed in the same sample. This is the case, for example, in the study of host-pathogen interactions. Specific guidelines for this type of experiments are important as the amount and characteristics of the RNA from both species might be different and hence a common analysis procedure might not work. The guidelines discusses experimental design, RNA extraction, preprocessing, differential expression and downstream analyses. Finally, the paper includes a section about scRNA-seq of multi-species samples.

I must congratulate the authors for putting together these fantastic guidelines for multi-species RNA-seq. The paper is well written, the guidelines are comprehensive, and I have enjoyed reading it. I think this is a paper that will be very welcomed by those working on host-pathogen interactions and other multi-species systems. I have, however, a number of recommendations that I believe should be incorporated in the paper to complement the quality of the work.

1. In page 6, targeted approaches are recommended for those cases where polyA RNA and rRNA depletion results in low RNA yields. I agree this is probably the only way to go. However, targeted capture might be expensive and knowledge about the species transcriptome is required, which might not be always available. It would be great if authors could elaborate more on the practical aspects of the targeted capture approach and its limitations.

2. Long-reads sequencing. Authors claim that long reads can be useful when the transcriptome of one of the species is not known or for the detection of isoforms. I agree with both statements, but I think they should also indicate that alternative isoforms are not generally expressed in microorganisms (they are in higher eukaryotes), and therefore this approach might not be as relevant for this purpose. I do agree that long reads are useful to capture long operons in bacteria, and the reference to the SMRT-Cappable-seq is pertinent. However, Iso-seq requires relatively high quantities of starting RNA and therefore may have limitations for a multi-species RNA-seq approach. Authors should include a discussion about these limitations in their paper.

3. At the section Quality control and read alignments, authors recommend both using different reads alignment algorithms and at the same time, mapping to the joint genomes of the multi-species. This sounds like a contradiction, since only one mapper can be used in the joint genome mapping, implying that one type of species would be better mapped than the other. Recommendations for a good working procedure should be indicated here.

4. Saturation curves. Perfect saturation is never reached, especially in higher eukaryotes. Some practical recommendations would be useful here. Authors cite the Tarazona et al.2011 paper in this section. They include a new transcript detection rate within the saturation curves that can be useful to establish a sequencing depth level at which it is no longer meaningful to increase sequencing.

5. The functional analysis of the multi-species data is particularly interesting, especially for those studying host-pathogens interactions. Some discussion of co-expression networks and pathway enrichment is provided. However, I miss additional directions as how to identify key molecules involved in these interactions. This might be a combination of experimental design and analysis choices. This is important, as the goal of many multi-species RNA-seq studies is to characterize pathogenic or symbiotic molecular interactions and a simple co-expression or enrichment analysis might not provide the right answers. I think a good addition to these guidelines would be to discuss papers where the unravelling of these interaction interfaces has been successfully addressed.

6. In the further directions section, I would like to see some discussion about how the field needs to evolve for the study of microbiome-host interactions using RNA-seq. Dysbiosis can lead to pathogenic relationships, but this is different from the classical host-pathogen interactions addressed in these guidelines. I can envision that additional challenges would apply when the gene expression of both the host and the associated microbiome needs to be simultaneously analyzed.

**Reviewer 2**

**Comments to author:**

In this manuscript, Chung and colleagues present a review of best practices for multi-species RNA-seq analysis. This is an important topic - these methods are becoming increasingly common, but there is very little in the way of standard practices when it comes to data generation and analysis, and many of the experimenters applying them have little in the way of statistical or computational training. Given the diversity of biological systems and questions which can be studied with the technique, it seems unlikely there will ever be a single standardized pipeline for their analysis. However practical guidance, particularly regarding quality control and possible data analyses and their assumption would be extremely valuable.

Unfortunately, the current manuscript has two major issues that substantially detract from its usefulness. The first is that the engagement with the multi-species RNA-seq literature is fairly shallow. For the majority of the sections in their manuscript, examples in the multi-species RNA-seq literature are available that could be used to illustrate principles, show practical results, and give the interested reader pointers to papers where the techniques have been applied. However, the only places in the manuscript where there are extended discussions of results from the literature refer primarily to the authors' own work (e.g. the discussion of target capture on lines 133-144; the discussion of BWA seed length on lines 192-197.) There is nothing wrong with warranted self-citations, but given the substantial multi-species RNA-seq literature it seems like more could be done to integrate interesting approaches and findings from other groups into the manuscript. I will provide specific examples and citations in my detailed comments below.

The second issue is that in several places strong opinions are presented as factual, sometimes without any supporting evidence, sometimes in the face of evidence to the contrary. This is probably most problematic in the section on differential expression analysis ("Prokaryotic differential expression analyses are inherently flawed when performed at the gene level."), but occurs with various minor points throughout the manuscript. Given that such a review, particular in Genome Biology, would likely become a major reference for non-experts and junior scientists interested in applying the technique it is absolutely critical that the distinction between established facts and the authors' speculation be made clear and the supporting evidence provided and appropriately referenced. Again, I will discuss specific examples in my detailed comments below.

While I believe these two issues are highly problematic for the current manuscript, I also believe the basic premise of the review is a good one, and could be salvaged with substantial rewriting. In this spirit I will discuss the specific issues I see in the manuscript in detail below, in the order in which they appear. I hope the authors will see this as constructive criticism and use it to improve the manuscript.

Detailed Comments:

Lines 81 - 88: There are a number of problems with the initial introduction of mRNA enrichment.

First, the statement that "preparations that lack any enrichment… fails (sic) to account for this potentially large difference in relative abundance of RNA" seems to imply that not enriching for the minor organism is either not possible or fundamentally wrong. However, a number of studies have taken exactly this approach, often by using a high multiplicity of infection (MOI), e.g. Mika-Gospodorz et al. Nat Comm. 2020, Aprianto et al. Genome Biology 2016, Baddal et al. mBio 2015. One can argue about the realism of such models and the generalizability of the results, but this is an argument about the model and not whether one can do dual RNA-seq on it. Additionally, some fairly realistic infection models do in fact lead to high enough bacterial load such that informative data can be generated with no enrichment for bacteria (e.g. Nuss et al. PNAS 2016 sequencing mouse Peyer's patches infected with Yersinia pseudotuberculosis, where only rRNA depletion was applied.)

The second problem is that the authors frame a wide range of techniques as being enrichment methods for the minor organism, including FACS and laser capture microdissection. While it is true that these methods will have the end result of enriching for minor organism RNA, these methods serve additional purposes, such as separating infected from bystander cells and ensuring a more homogenous population for the purposes of analyzing the host response and a more nuanced discussion seems appropriate.

Finally, the authors mention that "techniques with longer processing time need to incorporate steps to minimize further changes in the transcriptome", but do not suggest any methods for this. Westermann et al. Nature 2016 contains a thorough analysis of fixation techniques applied to dual RNA-seq samples in Extended Data Figure 1, though I would expect there would also be applicable results on fixation techniques in the recent single cell RNA-seq literature given the similar requirements for the methods.

Line 108, figure 1: The title of this section ("Standard enrichment methods for short-read sequencing"), surrounding discussion, and first half of figure 1 seem to imply that multi-species RNA-seq inherently requires construction of separate libraries with specialized depletions for the major and minor organisms. A large fraction of studies (if not the majority, at least in infection biology) have not done this, so I think both this section and the figure need to be restructured to accommodate this.

A deeper engagement with the literature here would probably be enlightening: quite a few papers have performed dual RNA-seq on infection time courses now, where the bacterial population is increasing over time. It should be possible to infer from these some rough recommendations about at which major:minor organism cell ratios (or such) enrichment becomes advisable.

Lines 125 - 127, the only rationale given for targeted capture is that one can not separate samples where there is not a difference in poly-A status of transcripts, i.e. in mixed eukaryote:eukaryote or prokaryote:prokaryote samples. However this ignores the authors own work showing that targeted capture is superior to poly-A and rRNA depletion in a Wolbachia:Brugia:Mosquito model (Chung et al. Sci. Rep 2018, ref 39) and can enable analysis of very low abundance pathogens (Betin et al. Sci Rep 2019, ref 38), as well as other work based on the same principle which the authors have not cited (e.g. Peterson et al. Mol Sys Biol 2019). A more nuanced discussion of the technique would be appreciated.

Additionally, throughout the manuscript, the authors repeatedly conflate rRNA depletion with enriching for minor organism RNA. Similarly to their discussion of FACS, while this does have the effect of enriching for minor organism RNA, this is not the sole, or in this case even primary, purpose. As some of the coauthors have published previously (e.g. Giannoukos et al. Genome Biology 2012) as much of 95% of the bacterial transcriptome consists of rRNA. This means even if major and minor organism RNA were present at equimolar ratios rRNA depletion would still be necessary. This should be clarified as it is a critical step in any RNA-seq experiment.

Line 145: in the section "enrichment methods for long-read sequencing", the authors seem ambivalent on whether long read sequencing could or should be applied to multi-species RNA-seq samples (e.g. it "may also have an advantage in the accurate identification of transcript isoforms"; unclear why SMRT-Cappable-seq, a method to identify primary transcripts, is the only approach the authors consider to look at bacteria; also unclear why the authors don't consider alternatives to poly-A tailing bacterial transcripts or alternative depletion/enrichment (e.g. target capture?) in making IsoSeq cDNA.) Finally, while figure 1 contains a reference to Oxford Nanopore direct RNA sequencing, as far as I can tell this is never referred to in the manuscript. This section could be clarified significantly, and if there is no clear message it could be easily removed.

Lines 195 - 197: The authors' extended discussion of their previous results in Robinson et al. Microb Genom 2017 seems odd. First, the major conclusion of that paper seems to be that combining the major and minor organism genomes in a single reference is important to prevent false positives, to quote: "This problem of read pairs aligning to both references was resolved, meaning they were mapped to the best position, by combining the Brugia and Wolbachia references into a single reference sequence"; "This problem of read pairs aligning to both references was resolved by combining the human and Plasmodium reference into one file." This is already mentioned on line 188 and is fine.

However, the recommendation for a seed length of 23 nt appears to mostly stem from the reduced CPU time, and an increase in specificity in the absence of one of the reference genomes. I was unable to find any reference in the paper to a greater number of reads mapping to the minor organism with a seed of 23 nt or increased sensitivity, again to quote in reference to mapping to the minor genome separately: "In the Brugia-Wolbachia dataset, reads mapping to the reference genome of the minority member (Wolbachia) decreased markedly from 3.3 to 2.9 % when increasing the seed length from 18 to 20 nt"; "Aligning the Plasmodium-human data to the Plasmodium reference or the Plasmodium-human combined reference led to a consistent percentage of read pairs mapping across seed length up to 30 nt". It would be strange if increasing seed length increased sensitivity, as a longer seed can only reduce the number of mapping locations considered by the aligner, and this seems to be what the authors have found - an increase in specificity in the special case of mapping to a single reference when other organisms are present in the sample.

Lines 206 - 208: It is not clear to me what the greater specificity of genome alignment has to do with assigning ambiguous fragments, maybe with the exception of something like LINE-element derived transcripts. A clearer explanation of the problems of transcriptome vs. genome alignment, and the advantages of model-based quantification tools would be warranted. The recent literature on selective alignment (e.g. Srivasatava et al. Genome Biology 2020) seems like it would be relevant.

Lines 218 - 221: It is a bit unclear what the authors have tested or shown in their manuscript apparently in revision at mSystems regarding the effects of gene vs. transcript quantification for operons. In the absence of either a published paper, a preprint, or at least a clear discussion of the data, I would recommend removing this.

Lines 230 - 232: It is unclear why using an alignment-free quantification tool on transcripts derived from CDS annotations would be substantially worse than aligning to the genome and quantifying CDSes with e.g. HTSeq. Again, Srivasatava et al. Genome Biology 2020 may be relevant. It is also unclear why the authors suggest these tools cannot be used when there are many highly overlapping transcripts; they were expressly developed to quantify isoforms where transcripts share substantial chunks of sequence (i.e. exons), see e.g. Bray et al Nat Meth 2016.

Line 234: The suggestion of using a saturation curve for quality control is a good one. However, it would probably make sense to illustrate this with an actual figure, as the text description is likely quite abstract to someone who has never seen one before. It is also not clear that a sample must achieve saturation to have "biological significance" as the authors suggest. In fact, one of the coauthors wrote a classic paper looking at exactly this issue, and reported that even very low coverage samples can sometimes be useful to detect things like the expression of key virulence factors (Haas et al. BMC Genomics 2012). Additionally, it is also not clear how the authors propose that one correct for batch effects in a fastq file (line 239), or how the analysis could be adjusted. I'm also not sure that throwing out samples is the best recommendation, particularly in cases where researcher may be working with samples that are practically or ethically difficult to reproduce (e.g. patient samples, samples from animal models). There is an extensive literature on accounting for poor quality samples and batch effects in RNA-seq data that would be relevant here and elsewhere in the manuscript that should probably be considered by the authors (e.g. Liu et al. NAR 2015, Risso et al. Nat. Biotech 2014, Leek NAR 2014).

Line 243: In addition to filtering low abundance transcripts, which is standard practice in RNA-seq analysis, the authors suggest filtering high abundance transcripts. This is not advice I have encountered previously, and it is unclear why one would do this, though I suppose there may be a reason for particular applications, like applying certain types of clustering algorithms. The reference the authors cite to support this advice (ref 70) evaluates a hemoglobin transcript depletion kit, used for similar reasons to rRNA depletion, as 50-80% of the blood transcriptome apparently consists of hemoglobin transcripts and this reduces coverage of more informative transcripts. This reference does not suggest that one should generally filter out high abundance transcripts computationally after sequencing, as the authors seem to think.

Lines 257 - 269: see previous comments regarding batch effects and when to exclude samples. This needs a more subtle and informed discussion.

Line 273: As far as I am aware TPMs were introduced in Li et al. Bioinformatics 2010 (ref 59), and this should be cited.

Lines 281 - 283: it would probably be worth giving the reader an intuition for what these normalization methods do, and whether and how they can fail.

Lines 287 - 296: The authors state that "Prokaryotic differential expression analyses are inherently flawed when performed at the gene level." This is quite an extraordinary claim, as it would imply that the hundreds, if not thousands of studies that have done this are in turn also inherently flawed. The reason given is that "differential expression tools treat each gene as an independent variable when estimating variation". The authors seem to have confused notions of independent transcription with statistical independence. To be pedantic on this point, it seems unlikely that any two genes' expression values chosen at random in any organism are statistically independent from one another: shared regulation, cell state, and environment will all induce some degree of statistical dependence. The empirical Bayes methods used for estimating the variance of expression levels were initially proposed in part explicitly because they are robust to violations of independence. One can see the authors who first introduced these techniques considered this, e.g.: "The normality and independence assumptions we make are not meant to be taken literally, rather, they are to be regarded as devices leading to a formula which we believe improves upon the average and the t statistic in this context." (Lönnstedt and Speed, Stat Sin. 2002); "The hierarchical model defined in the next section will assume that the estimators ... from different genes are independent. Although this is not necessarily a realistic assumption, the methodology which will be derived makes qualitative sense even when the genes are dependent, as they likely will be for data from actual microarray experiments." (Smyth, Stat Appl Genet Mol Biol 2004).

It is possible that the physical linkage between genes in transcripts is somehow a special form of dependence that breaks the empirical Bayes heuristic programs like DESeq and edgeR use. Potentially one could investigate this with simulations. However, the authors provide no evidence for this; the only references in this section (76 and 77) have to do with predicting operon structure, not their effect on differential expression analysis. While I agree with the authors that the objects we ultimately want to be quantifying are individual transcripts, and doing this may have some effects on the way we interpret differential expression at the margins, I see no reason to think that operon structure makes differential expression analysis in prokaryotes any less valid than differential expression analysis in eukaryotes, let alone "inherently flawed."

Line 297, section "Clustering genes by expression patterns": The authors do not explain what a Z-score is, why it is applied, and moreover it is unclear why they imply Z-scores are the only data transformation on which clustering could be performed. An obvious alternative if one were doing, say, an infection time-course might be log fold-changes from a laboratory-grown inoculum. This would have the same effect as a Z-score (setting rows to the same scale), but likely be more informative as the fold-changes in this case have a clear biological interpretation. Again, figures illustrating these analyses would probably be more useful than, or could be shown in addition to, flowcharts.

The second issue with this section is that it does not engage at all with the multi-species RNA-seq literature, or the network analysis literature really for that matter. Given that there is a whole field of network analysis approaches it's odd that only WGCNA and EPIG-Seq are mentioned. Additionally, a number of papers have performed the sorts of analyses the authors are suggesting and have been ignored. In fact, one dual RNA-seq paper (Grote et al. PLoS NTDs 2017) has applied exactly the WGCNA method suggested here. Off the top of my head several others have applied alternative clustering methods (e.g. Westermann et al. Nature 2016; Griesenauer et al. mBio 2019). I am sure a comprehensive literature review would turn up more. Again, this could be a great source for figures and examples illustrating what exactly one can get from these analyses.

Line 329, section "Functional analyses": the authors do not discriminate between gene set analysis methods (e.g. DAVID) and databases (e.g. KEGG), and there isn't much of a discussion of what it is these methods and databases actually do or are good for.

Line 338: unclear why there is a sub-heading "Other applications" when the only other application is single cell RNA-seq.

Lines 347 - 351: Could the authors explain how they might envision one separating e.g. intracellular bacteria from host cells for single cell applications? This section has some interesting ideas, but could use some development and appropriate references.

Figures: generally, the workflow figures are useful, though it would probably be advisable to add panels showing examples for the different analysis steps and methods the authors suggest, and what could be extracted from them (e.g. saturation curves, clustering methods). Also, at several points tools are suggested in the figures that are never discussed in the text (e.g. NGSQC, several of the de novo transcriptome assembly methods) and vice versa (e.g. cutadapt, none of the model based quantification tools like RSEM). Also, the figure quality is quite low and pixelated, though this may be an artifact of the article submission system.

**Authors Response**

**Point-by-point responses to the reviewers’ comments:**

Reviewer 1:

Chung et at. present a guidelines paper for RNA-seq analysis in multi-species experiments, i.e. with the RNA from more than one species is analyzed in the same sample. This is the case, for example, in the study of host-pathogen interactions. Specific guidelines for this type of experiments are important as the amount and characteristics of the RNA from both species might be different and hence a common analysis procedure might not work. The guidelines discusses experimental design, RNA extraction, preprocessing, differential expression and downstream analyses. Finally, the paper includes a section about scRNA-seq of multi-species samples.

I must congratulate the authors for putting together these fantastic guidelines for multi-species RNA-seq. The paper is well written, the guidelines are comprehensive, and I have enjoyed reading it. I think this is a paper that will be very welcomed by those working on host-pathogen interactions and other multi-species systems. I have, however, a number of recommendations that I believe should be incorporated in the paper to complement the quality of the work.

*Thank you for your positive comments and on these recommendations. Our responses to them are outlined below with line numbers.*

1. In page 6, targeted approaches are recommended for those cases where polyA RNA and rRNA depletion results in low RNA yields. I agree this is probably the only way to go. However, targeted capture might be expensive and knowledge about the species transcriptome is required, which might not be always available. It would be great if authors could elaborate more on the practical aspects of the targeted capture approach and its limitations.

*We have added text to elaborate more on the practical aspects of the targeted capture*

*approach and its limitations on lines 132-160.*

2. Long-reads sequencing. Authors claim that long reads can be useful when the transcriptome of one of the species is not known or for the detection of isoforms. I agree with both statements, but I think they should also indicate that alternative isoforms are not generally expressed in microorganisms (they are in higher eukaryotes), and therefore this approach might not be as relevant for this purpose. I do agree that long reads are useful to capture long operons in bacteria, and the reference to the SMRT-Cappable-seq is pertinent. However, Iso-seq requires relatively high quantities of starting RNA and therefore may have limitations for a multi-species RNA-seq approach. Authors should include a discussion about these limitations in their paper.

*We added an additional reference and text that describes what is known about*

*alternate transcription in at least prokaryote. We also added text as to the limitations on*

*lines 171-172 and 182-190.*

3. At the section Quality control and read alignments, authors recommend both using different reads alignment algorithms and at the same time, mapping to the joint genomes of the multi-species. This sounds like a contradiction, since only one mapper can be used in the joint genome mapping, implying that one type of species would be better mapped than the other. Recommendations for a good working procedure should be indicated here.

*We clarified that when one organism in the system has splicing that a splice-aware*

*aligner should be used for all on line 221.*

4. Saturation curves. Perfect saturation is never reached, especially in higher eukaryotes. Some practical recommendations would be useful here. Authors cite the Tarazona et al.2011 paper in this section. They include a new transcript detection rate within the saturation curves that can be useful to establish a sequencing depth level at which it is no longer meaningful to increase sequencing.

*We are unsure what the reviewer meant by “They include a new transcript detection*

*rate within the saturation curves that can be useful to establish a sequencing depth*

*level at which it is no longer meaningful to increase sequencing.” While the Tarazona*

*paper is a good reference for curves, we don’t see a method or results like the reviewer*

*suggests. Tarazona emphasizes balanced sequencing depth, which is frequently not*

*possible in these studies. They also describe NOISeq, but that does not seem to*

*address this issue. However, per the suggestions of the other reviewer, we did add a*

*figure and further text to this section on lines 275-290.*

5. The functional analysis of the multi-species data is particularly interesting, especially for those studying host-pathogens interactions. Some discussion of co-expression networks and pathway enrichment is provided. However, I miss additional directions as how to identify key molecules involved in these interactions. This might be a combination of experimental design and analysis choices. This is important, as the goal of many multi-species RNA-seq studies is to characterize pathogenic or symbiotic molecular interactions and a simple co-expression or enrichment analysis might not provide the right answers. I think a good addition to these guidelines would be to discuss papers where the unravelling of these interaction interfaces has been successfully addressed.

*We have added some examples of how this has been applied to several sections*

*including including on lines 408-413, lines 422-426, and lines 439-443.*

6. In the further directions section, I would like to see some discussion about how the field needs to evolve for the study of microbiome-host interactions using RNA-seq. Dysbiosis can lead to pathogenic relationships, but this is different from the classical host-pathogen interactions addressed in these guidelines. I can envision that additional challenges would apply when the gene expression of both the host and the associated microbiome needs to be simultaneously analyzed.

*We have added a short metagenomics section on lines 480-506. While not a complete*

*review on the topic, it introduces the topic.*

Reviewer 2:

In this manuscript, Chung and colleagues present a review of best practices for multi-species RNA-seq analysis. This is an important topic - these methods are becoming increasingly common, but there is very little in the way of standard practices when it comes to data generation and analysis, and many of the experimenters applying them have little in the way of statistical or computational training. Given the diversity of biological systems and questions which can be studied with the technique, it seems unlikely there will ever be a single standardized pipeline for their analysis. However practical guidance, particularly regarding quality control and possible data analyses and their assumption would be extremely valuable.

Unfortunately, the current manuscript has two major issues that substantially detract from its usefulness. The first is that the engagement with the multi-species RNA-seq literature is fairly shallow. For the majority of the sections in their manuscript, examples in the multi-species RNA-seq literature are available that could be used to illustrate principles, show practical results, and give the interested reader pointers to papers where the techniques have been applied. However, the only places in the manuscript where there are extended discussions of results from the literature refer primarily to the authors' own work (e.g. the discussion of target capture on lines 133-144; the discussion of BWA seed length on lines 192-197.) There is nothing wrong with warranted self-citations, but given the substantial multi-species RNA-seq literature it seems like more could be done to integrate interesting approaches and findings from other groups into the manuscript. I will provide specific examples and citations in my detailed comments below.

The second issue is that in several places strong opinions are presented as factual, sometimes without any supporting evidence, sometimes in the face of evidence to the contrary. This is probably most problematic in the section on differential expression analysis ("Prokaryotic differential expression analyses are inherently flawed when performed at the gene level."), but occurs with various minor points throughout the manuscript. Given that such a review, particular in Genome Biology, would likely become a major reference for non-experts and junior scientists interested in applying the technique it is absolutely critical that the distinction between established facts and the authors' speculation be made clear and the supporting evidence provided and appropriately referenced. Again, I will discuss specific examples in my detailed comments below.

While I believe these two issues are highly problematic for the current manuscript, I also believe the basic premise of the review is a good one, and could be salvaged with substantial rewriting. In this spirit I will discuss the specific issues I see in the manuscript in detail below, in the order in which they appear. I hope the authors will see this as constructive criticism and use it to improve the manuscript.

*Thank you for your support and constructive criticism that has been used to improve the manuscript. Our responses are outlined below with line numbers. We have also numbered the reviewer’s comments to help aid us in revisions and responses.*

Detailed Comments:

Lines 81 - 88: There are a number of problems with the initial introduction of mRNA enrichment.

First, the statement that "preparations that lack any enrichment… fails (sic) to account for this potentially large difference in relative abundance of RNA" seems to imply that not enriching for the minor organism is either not possible or fundamentally wrong. However, a number of studies have taken exactly this approach, often by using a high multiplicity of infection (MOI), e.g. Mika-Gospodorz et al. Nat Comm. 2020, Aprianto et al. Genome Biology 2016, Baddal et al. mBio 2015. One can argue about the realism of such models and the generalizability of the results, but this is an argument about the model and not whether one can do dual RNA-seq on it. Additionally, some fairly realistic infection models do in fact lead to high enough bacterial load such that informative data can be generated with no enrichment for bacteria (e.g. Nuss et al. PNAS 2016 sequencing mouse Peyer's patches infected with Yersinia pseudotuberculosis, where only rRNA depletion was applied.)

*This text was meant to introduce the subsequent sentences. We deemed it confusing and unnecessary, and we deleted it from the manuscript.*

The second problem is that the authors frame a wide range of techniques as being enrichment methods for the minor organism, including FACS and laser capture microdissection. While it is true that these methods will have the end result of enriching for minor organism RNA, these methods serve additional purposes, such as separating infected from bystander cells and ensuring a more homogenous population for the purposes of analyzing the host response and a more nuanced discussion seems appropriate.

*Added the requested text and rephrased the sentence for clarity on line 85.*

Finally, the authors mention that "techniques with longer processing time need to incorporate steps to minimize further changes in the transcriptome", but do not suggest any methods for this. Westermann et al. Nature 2016 contains a thorough analysis of fixation techniques applied to dual RNA-seq samples in Extended Data Figure 1, though I would expect there would also be applicable results on fixation techniques in the recent single cell RNA-seq literature given the similar requirements for the methods.

*Fixation conditions likely depend largely on the system. The paper the reviewer mentioned is only for Salmonella, and it isn’t clear those results will necessarily be generalizable. On line 89, we include a reference to that paper, but leave the text more open-ended than we interpret the reviewer’s suggestion to mean.*

Line 108, figure 1: The title of this section ("Standard enrichment methods for short-read sequencing"), surrounding discussion, and first half of figure 1 seem to imply that multi-species RNA-seq inherently requires construction of separate libraries with specialized depletions for the major and minor organisms. A large fraction of studies (if not the majority, at least in infection biology) have not done this, so I think both this section and the figure need to be restructured to accommodate this.

*We changed the title of this section on line 110 and replaced all the text for “standard enrichment” to instead refer to rRNA and polyA-RNA and enrichment strategies. The figure has also been modified to add arrows for only polyA enrichment or only rRNA depletion.*

A deeper engagement with the literature here would probably be enlightening: quite a few papers have performed dual RNA-seq on infection time courses now, where the bacterial population is increasing over time. It should be possible to infer from these some rough recommendations about at which major:minor organism cell ratios (or such) enrichment becomes advisable.

*As highlighted in this section, it depends a lot upon the system, particularly the size the transcriptome. Therefore, we think it ill-advise to recommend major:minor cell ratios. Instead we propose a more data driven approach.*

Lines 125 - 127, the only rationale given for targeted capture is that one can not separate samples where there is not a difference in poly-A status of transcripts, i.e. in mixed eukaryote:eukaryote or prokaryote:prokaryote samples. However this ignores the authors own work showing that targeted capture is superior to poly-A and rRNA depletion in a Wolbachia:Brugia:Mosquito model (Chung et al. Sci. Rep 2018, ref 39) and can enable analysis of very low abundance pathogens (Betin et al. Sci Rep 2019, ref 38), as well as other work based on the same principle which the authors have not cited (e.g. Peterson et al. Mol Sys Biol 2019). A more nuanced discussion of the technique would be appreciated.

*We provided further details for the Chung et al. paper on lines 146-151 and the reference as it has now been published.*

Additionally, throughout the manuscript, the authors repeatedly conflate rRNA depletion with enriching for minor organism RNA. Similarly to their discussion of FACS, while this does have the effect of enriching for minor organism RNA, this is not the sole, or in this case even primary, purpose. As some of the coauthors have published previously (e.g. Giannoukos et al. Genome Biology 2012) as much of 95% of the bacterial transcriptome consists of rRNA. This means even if major and minor organism RNA were present at equimolar ratios rRNA depletion would still be necessary. This should be clarified as it is a critical step in any RNA-seq experiment.

*The text in this section starting on line 110 has been modified to address this comment while still also applying to eukaryote-eukaryote systems. However, some dual-species transcriptomics experiments have been done without rRNA depletion, so we do not feel it is critical for all systems. Eukaryote-eukaryote systems would not require it. But even some endosymbiont systems do not as the host has a very low amount of rRNA. We would like this to be broadly focused than for instance, humanbacteria.*

Line 145: in the section "enrichment methods for long-read sequencing", the authors seem ambivalent on whether long read sequencing could or should be applied to multi-species RNA-seq samples (e.g. it "may also have an advantage in the accurate identification of transcript isoforms"; unclear why SMRT-Cappable-seq, a method to identify primary transcripts, is the only approach the authors consider to look at bacteria; also unclear why the authors don't consider alternatives to poly-A tailing bacterial transcripts or alternative depletion/enrichment (e.g. target capture?) in making IsoSeq cDNA.) Finally, while figure 1 contains a reference to Oxford Nanopore direct RNA sequencing, as far as I can tell this is never referred to in the manuscript. This section could be clarified significantly, and if there is no clear message it could be easily removed.

*I don’t know of an alternative to polyA tailing bacterial transcripts to get full length transcripts to propose. One could polyG tail, or ligate an adapter, but I’m not sure either of these are advantageous, and both seem derivative. The only other advancements I know of are the unpublished work of others that is their IP. However, we tried to clarify this section on lines 161--190, giving a stronger message and made the requested changes to figure 1.*

Lines 195 - 197: The authors' extended discussion of their previous results in Robinson et al. Microb Genom 2017 seems odd. First, the major conclusion of that paper seems to be that combining the major and minor organism genomes in a single reference is important to prevent false positives, to quote: "This problem of read pairs aligning to both references was resolved, meaning they were mapped to the best position, by combining the Brugia and Wolbachia references into a single reference sequence"; "This problem of read pairs aligning to both references was resolved by combining the human and Plasmodium reference into one file." This is already mentioned on line 188 and is fine.

*We clarified that this is important when the organisms cannot be merged at mapping on lines 225-230. This can still be an issue when working with multiple large genomes, like two large eukaryotic genomics.*

However, the recommendation for a seed length of 23 nt appears to mostly stem from the reduced CPU time, and an increase in specificity in the absence of one of the reference genomes. I was unable to find any reference in the paper to a greater number of reads mapping to the minor organism with a seed of 23 nt or increased sensitivity, again to quote in reference to mapping to the minor genome separately: "In the Brugia-Wolbachia dataset, reads mapping to the reference genome of the minority member (Wolbachia) decreased markedly from 3.3 to 2.9 % when increasing the seed length from 18 to 20 nt"; "Aligning the Plasmodium-human data to the Plasmodium reference or the Plasmodium-human combined reference led to a consistent percentage of read pairs mapping across seed length up to 30 nt". It would be strange if increasing seed length increased sensitivity, as a longer seed can only reduce the number of mapping locations considered by the aligner, and this seems to be what the authors have found - an increase in specificity in the special case of mapping to a single reference when other organisms are present in the sample.

*Sensitivity was used instead of specificity, but most of this was deleted as part of the edits for the previous comment.*

Lines 206 - 208: It is not clear to me what the greater specificity of genome alignment has to do with assigning ambiguous fragments, maybe with the exception of something like LINE-element derived transcripts. A clearer explanation of the problems of transcriptome vs. genome alignment, and the advantages of model-based quantification tools would be warranted. The recent literature on selective alignment (e.g. Srivasatava et al. Genome Biology 2020) seems like it would be relevant.

*Many organisms only have CDSs annotated, which means that suboptimal mappings occur when only mapping to the transcriptome. This is well described in Srivasatava, which is now referenced, along with an expansion of the text on lines 245-247.*

Lines 218 - 221: It is a bit unclear what the authors have tested or shown in their manuscript apparently in revision at mSystems regarding the effects of gene vs. transcript quantification for operons. In the absence of either a published paper, a preprint, or at least a clear discussion of the data, I would recommend removing this.

*Many organisms only have CDSs annotated, which means that suboptimal mappings occur when only mapping to the transcriptome. This is well described in Srivasatava, which is now referenced, along with an expansion of the text on lines 252-257.*

Lines 230 - 232: It is unclear why using an alignment-free quantification tool on transcripts derived from CDS annotations would be substantially worse than aligning to the genome and quantifying CDSes with e.g. HTSeq. Again, Srivasatava et al. Genome Biology 2020 may be relevant. It is also unclear why the authors suggest these tools cannot be used when there are many highly overlapping transcripts; they were expressly developed to quantify isoforms where transcripts share substantial chunks of sequence (i.e. exons), see e.g. Bray et al Nat Meth 2016.

*We have included a reference to Srivasatava et al. Genome Biology 2020. With respect to why these tools may not work on prokaryotes, in the long-read data we have it is unlikely these algorithms could disentangle transcript quantification at this time. The transcripts can be very long with multiple internal starts and stops, and there is no differential splicing to help disentangle it. We frame this as “more testing needs to be done” as we don’t have a reference and also do not feel it is appropriate to include new laboratory data in a review.*

Line 234: The suggestion of using a saturation curve for quality control is a good one. However, it would probably make sense to illustrate this with an actual figure, as the text description is likely quite abstract to someone who has never seen one before. It is also not clear that a sample must achieve saturation to have "biological significance" as the authors suggest. In fact, one of the coauthors wrote a classic paper looking at exactly this issue, and reported that even very low coverage samples can sometimes be useful to detect things like the expression of key virulence factors (Haas et al. BMC Genomics 2012). Additionally, it is also not clear how the authors propose that one correct for batch effects in a fastq file (line 239), or how the analysis could be adjusted. I'm also not sure that throwing out samples is the best recommendation, particularly in cases where researcher may be working with samples that are practically or ethically difficult to reproduce (e.g. patient samples, samples from animal models). There is an extensive literature on accounting for poor quality samples and batch effects in RNA-seq data that would be relevant here and elsewhere in the manuscript that should probably be considered by the authors (e.g. Liu et al. NAR 2015, Risso et al. Nat. Biotech 2014, Leek NAR 2014).

*We added more details about saturation curves including an additional figure and more text on interpreting saturation curves on lines 275-290. We are not recommending that a researcher necessarily discard samples, but suggest it as an option, and clearly state that one alternative is that ”the analysis should be adjusted accordingly”. We have reworded to ensure that is clear. We have also provided a reference to a successful such analysis where a sample was removed and one where an alternate method was used. In addition, a call out is made to the new section that was added to discuss batch effects that includes the references mentioned as well as a few others.*

Line 243: In addition to filtering low abundance transcripts, which is standard practice in RNA-seq analysis, the authors suggest filtering high abundance transcripts. This is not advice I have encountered previously, and it is unclear why one would do this, though I suppose there may be a reason for particular applications, like applying certain types of clustering algorithms. The reference the authors cite to support this advice (ref 70) evaluates a hemoglobin transcript depletion kit, used for similar reasons to rRNA depletion, as 50-80% of the blood transcriptome apparently consists of hemoglobin transcripts and this reduces coverage of more informative transcripts. This reference does not suggest that one should generally filter out high abundance transcripts computationally after sequencing, as the authors seem to think.

*We provide text and four additional references to Wolbachia papers that all have this issue on lines 304-306. We have also had to remove hemoglobin transcripts in an unpublished paper for a collaborator who did work on blood and did not deplete hemoglobin transcripts experimentally.*

Lines 257 - 269: see previous comments regarding batch effects and when to exclude samples. This needs a more subtle and informed discussion.

*Reworded on lines 311-312 to direct to the new section on batch effects on lines 331- 340.*

Line 273: As far as I am aware TPMs were introduced in Li et al. Bioinformatics 2010 (ref 59), and this should be cited.

*We added a reference to TPM in Li et al. 2011 BMC Bioinformatics on line 325.*

Lines 281 - 283: it would probably be worth giving the reader an intuition for what these normalization methods do, and whether and how they can fail.

*We have briefly described these methods and included a reference to a review on lines 342-344.*

Lines 287 - 296: The authors state that "Prokaryotic differential expression analyses are inherently flawed when performed at the gene level." This is quite an extraordinary claim, as it would imply that the hundreds, if not thousands of studies that have done this are in turn also inherently flawed. The reason given is that "differential expression tools treat each gene as an independent variable when estimating variation". The authors seem to have confused notions of independent transcription with statistical independence. To be pedantic on this point, it seems unlikely that any two genes' expression values chosen at random in any organism are statistically independent from one another: shared regulation, cell state, and environment will all induce some degree of statistical dependence. The empirical Bayes methods used for estimating the variance of expression levels were initially proposed in part explicitly because they are robust to violations of independence. One can see the authors who first introduced these techniques considered this, e.g.: "The normality and independence assumptions we make are not meant to be taken literally, rather, they are to be regarded as devices leading to a formula which we believe improves upon the average and the t statistic in this context." (Lönnstedt and Speed, Stat Sin. 2002); "The hierarchical model defined in the next section will assume that the estimators ... from different genes are independent. Although this is not necessarily a realistic assumption, the methodology which will be derived makes qualitative sense even when the genes are dependent, as they likely will be for data from actual microarray experiments." (Smyth, Stat Appl Genet Mol Biol 2004).

*These papers discuss independence in a different manner, that one gene going up has to be met with all others going down by the same amount given that relative abundance to one another is the approach. That is different than the lack of independence associated with operons where all the values should have the same value, which is still problematic. None of the papers address that issue.*

It is possible that the physical linkage between genes in transcripts is somehow a special form of dependence that breaks the empirical Bayes heuristic programs like DESeq and edgeR use. Potentially one could investigate this with simulations. However, the authors provide no evidence for this; the only references in this section (76 and 77) have to do with predicting operon structure, not their effect on differential expression analysis. While I agree with the authors that the objects we ultimately want to be quantifying are individual transcripts, and doing this may have some effects on the way we interpret differential expression at the margins, I see no reason to think that operon structure makes differential expression analysis in prokaryotes any less valid than differential expression analysis in eukaryotes, let alone "inherently flawed."

*We have added the simulations to the review and provide evidence that this is a significant problem as described on lines 352-379. We became aware of this problem in our own studies along with the problems solved with FADU—both of which were identified through examination of unusual results with genes in known operons.*

Line 297, section "Clustering genes by expression patterns": The authors do not explain what a Z-score is, why it is applied, and moreover it is unclear why they imply Z-scores are the only data transformation on which clustering could be performed. An obvious alternative if one were doing, say, an infection time-course might be log fold-changes from a laboratory-grown inoculum. This would have the same effect as a Z-score (setting rows to the same scale), but likely be more informative as the fold-changes in this case have a clear biological interpretation. Again, figures illustrating these analyses would probably be more useful than, or could be shown in addition to, flowcharts.

*We re-worded the section on lines 381-390, providing more options and explaining the utility of z-scores for reference-less comparisons.*

The second issue with this section is that it does not engage at all with the multi-species RNA-seq literature, or the network analysis literature really for that matter. Given that there is a whole field of network analysis approaches it's odd that only WGCNA and EPIG-Seq are mentioned. Additionally, a number of papers have performed the sorts of analyses the authors are suggesting and have been ignored. In fact, one dual RNA-seq paper (Grote et al. PLoS NTDs 2017) has applied exactly the WGCNA method suggested here. Off the top of my head several others have applied alternative clustering methods (e.g. Westermann et al. Nature 2016; Griesenauer et al. mBio 2019). I am sure a comprehensive literature review would turn up more. Again, this could be a great source for figures and examples illustrating what exactly one can get from these analyses.

*We have added these references and more to ensure that each section ends with a bit of biology, including lines 408-413, lines 422-426, and lines 439-443.*

Line 329, section "Functional analyses": the authors do not discriminate between gene set analysis methods (e.g. DAVID) and databases (e.g. KEGG), and there isn't much of a discussion of what it is these methods and databases actually do or are good for.

*This section starting on line 427 has been refined to address the reviewer’s concerns.*

Line 338: unclear why there is a sub-heading "Other applications" when the only other application is single cell RNA-seq.

*Metagenomics was added to this section so this is no longer an issue.*

Lines 347 - 351: Could the authors explain how they might envision one separating e.g. intracellular bacteria from host cells for single cell applications? This section has some interesting ideas, but could use some development and appropriate references.

*Added text to discuss challenges that intracellular bacteria may pose, but also some of the opportunities on lines 458-460.*

Figures: generally, the workflow figures are useful, though it would probably be advisable to add panels showing examples for the different analysis steps and methods the authors suggest, and what could be extracted from them (e.g. saturation curves, clustering methods). Also, at several points tools are suggested in the figures that are never discussed in the text (e.g. NGSQC, several of the de novo transcriptome assembly methods) and vice versa (e.g. cutadapt, none of the model based quantification tools like RSEM). Also, the figure quality is quite low and pixelated, though this may be an artifact of the article submission system.

*The figures have been updated given the reviewer’s suggestion.*

**Second round of review**

**Reviewer 1**

My points of review have been successfully addressed.

However, the authors did not understand my comment about saturation curves. Please look at the saturation plots in Figure 1 in the Tarazona et al. 2011 paper. Vertical bars represent the number of newly detected genes per million additional reads, indicated in the right Y axis. I found this to be more informative than saturation curves as appreciation of the curve becoming flat or not can be modified simply by changing the scale of the left Y axes. The rate of new detections is a more quantitative measure.

**Reviewer 2**

Chung and colleagues have revised their review of best practices for multi-species RNA-seq analysis. While it is improved, there are still a number of outstanding issues that need to be corrected. I would stress that my main concern is that this will be read by students and non-experts, and really must be accurate and clear so as not to propagate confusion.

Major issues:

Major issue 1:

The statement that "Genes with … high abundance should be excluded before downstream analyses" remains on line 292. After reading this section multiple times, my best guess at the authors' rationale for this is one or two highly variable, high abundance genes might make automatic color scale ranges in heatmaps so large that it is difficult to see variation in expression of other genes. This is not a terribly convincing reason, seeing as it can be easily solved by adjusting the color scale range as is commonly done.

As I stated in my review of the previous version, there may be particular analyses where this makes sense to do, but if so the authors need to explain this, clearly. To make this crystal clear, these are the problems with this statement and the framing in this section:

1. The positioning in the paper is such that it seems like the authors are implying high-abundance genes should be excluded before *all* downstream analyses, for instance differential expression analysis. There is *no* basis for that particular recommendation. It is possible to read this as only applying to cluster analyses, but even then it is not clear to me that this is really necessary in most cases besides for presentation purposes, and an article explicitly billing itself as setting out "best practices" should not contain this kind of ambiguity about such a basic issue.

2. The authors continue to confuse the reasons for experimental hemoglobin transcript depletion in reference 88. This depletion is to allow better quantification of the rest of the transcriptome, for the same reasons as one would do rRNA depletion, not because having a lot of hemoglobin transcript reads screws up downstream data analysis.

3. The authors cite four papers, including two of their own, to support this practice. I have gone through these papers and again find no support for this. I cannot pretend to know what exactly the authors are referring to with these papers, but I would note that according to their methods sections they do not filter high abundance genes before their own differential expression analysis. The do indeed not include 6S in their cluster analyses, but this is because they only perform cluster analysis on protein coding genes.

The closest I could find to an actual statement on the issue these papers were cited to support was in reference 41 where the authors write "this analysis of global gene regulation was conducted only on coding sequences (CDSs), precluding observation of differentially expressed noncoding RNAs, like the 6S RNA". Reference 70 also only analyzes coding genes as best as I can tell, and the only mention of 6S I could find is in a supplementary figure. The two references not by the authors of this review also do not seem to support this. Reference 89 makes no mention of 6S that I could find. Reference 90 does talk a bit about 6S/ssrS, but this seems to be because they have explicitly included it in their analyses and suggest it may be an important regulator for the process they are studying.

Major Issue 2:

The authors continue to state that "treating every gene as an independent variable for bacterial samples leads to errors in variance estimations", on line 355. In their response to my previous comment on the issue, the authors state "these papers [that I cited] discuss independence in a different manner, that one gene going up has to be met with all others going down by the same amount given that relative abundance to one another is the approach." This is false. Lönnstedt and Speed 2002 and Smyth 2004 introduced the empirical Bayes method for estimating the variance in expression data, which is what the authors claim is being confounded by dependence between gene expression level measurements. If the authors would prefer an explicit reference for RNA-seq data I would direct them to Robinson and Smyth, Biostatistics 2008, which states: "We assume that all tags are independent for the purposes of estimation and inference. This is not strictly true since some sets of genes are involved in related biological functions and are inherently coregulated in their expression levels. However, our primary concern is getting an unbiased estimate of the dispersion parameter and consistency of the estimator is unaffected by this correlation."

I would like to reiterate here that I agree with the authors that the ultimate object of quantification should be transcripts, since this is what RNA-seq is measuring. What I object to is ascribing the problem with not doing this to some vague statistical issue that likely does not exist.

The authors now include a simulation study the authors claim shows that modeling coding sequences as independent variables leads to problems in variance estimation. While the details of this simulation study are inadequately described (e.g. what were the effect sizes? Are overlapping transcript models included? If so, how are they dealt with? How many reads were generated?), I see no reason to doubt the basic conclusion that one could more accurately detect differential expression with long transcripts than shorter genes. This does not imply anything about the variance estimation procedure, as this simulation conflates *at least* three separate issues: different accuracy in quantification against different gene models, differences in the amount of data for the different objects of quantification, and then possibly issues in the statistical model underlying the analysis.

The simplest explanation for these simulation results is that as differential expression tools work on count data, and longer transcripts generate more reads, it will simply be easier to detect smaller changes in expression for longer transcripts as there is more data. This is a well-known bias in RNA-seq data (see Young et al. "Gene ontology analysis for RNA-seq: accounting for selection bias" Genome Biology 2010). To show that this is a problem with variance estimation would require a much more involved simulation where the dispersion parameter is directly measured and manipulated, but to be honest this doesn't make a lot of sense to even attempt unless the authors are really committed to showing dependence relationships affect variance estimation.

Minor issues:

From my previous comments: 2-17 Line 273: As far as I am aware TPMs were introduced in Li et al. Bioinformatics 2010 (ref 59), and this should be cited.

Author reply: We added a reference to TPM in Li et al. 2011 BMC Bioinformatics on line 325.

The correct reference is Li et al. "RNA-seq gene expression estimation with read mapping uncertainty" Bioinformatics 2010. Feel free to cite Li et al BMC Bioinformatics 2011 as well, though. Neither is currently cited on line 325 (ref 92 remains Wagner et al Theory Biosci 2013).

Line 48: Reference 15 did not coin the term "dual-species transcriptomics", it coined the term "dual RNA-seq". The phrase "dual-species transcriptomics" is not used in the article.

Line 88: there is a disconnect between the statement in the paper that "techniques with longer processing time need to incorporate steps to minimize further changes in the transcriptome", and their insistence in their reply to reviewers that because only a single study has done this it is impossible to give any reasonable advice or leads for further research. Is it necessary or not? If it is necessary more than one study must have done this? As I previously wrote, surely there is some literature around this it is possible to extrapolate from, possibly in the single-cell or meta'omics literature? Westermann et al. PLoS Pathogens 2017 for instance includes a box on this issue, though is three years out of date now and was written before single-cell analysis really exploded.

Line 173 and reply to reviewers: The authors seem to be under the impression that transcriptional complexity in bacteria is new. Studies looking at this transcriptome-wide date to at least 2009 (e.g. Güell et al., Science 2009; for one review see Wage and Grainger, "Pervasive transcription: illuminating the dark matter of bacterial transcriptomes" Nature Reviews Microbiology 2014, there are many others).

Line 272: Do the authors mean exons?

Line 286: what is "biological significance"?

Line 287: I still do not understand how one would account for batch effects in a fastq file, and certainly not how one would merge them after. To be clear, batch effects are generally assessed on the *count* level, which is after reads have already been mapped and quantified, and I'm not sure I even understand what it would mean to propagate this back to the fastq files and merge them after.

Line 289: The authors continue to say in regard to unsaturated libraries they should be discarded or "the analysis should be adjusted accordingly" and cite one of their own papers with no further explanation. This paper does seem to be possibly relevant (it just looks at highly expressed genes), but it is not obvious and took a fairly close reading to understand why this paper was being cited (the final section of the results discusses this a bit). Surely a review of best practices should provide a little more concrete advice.

Line 289: prevented -> presented

Line 350: if the authors are going to bring up sleuth and bootstrap estimates, it would be good to specify what this is for, as they are used to estimate technical variation in quantification and not for count normalization as the placement in this paragraph implies. If so, it may also be worth including the swish paper here (Zhu et al NAR 2019).

References: three references I mentioned in my previous review (30-32) are in the reference list, along with reference 33, and never cited. I don't particularly care if they're cited or not, but this seems to be a formatting error.

**Authors Response**

**Point-by-point responses to the reviewers’ comments:**

Reviewer 1:

My points of review have been successfully addressed.

However, the authors did not understand my comment about saturation curves. Please look at the saturation plots in Figure 1 in the Tarazona et al. 2011 paper. Vertical bars represent the number of newly detected genes per million additional reads, indicated in the right Y axis. I found this to be more informative than saturation curves as appreciation of the curve becoming flat or not can be modified simply by changing the scale of the left Y axes. The rate of new detections is a more quantitative measure.

*Got it. Thanks for the additional clarification. We have added text on this and a citation to Tarazona. We tried to implement that approach for inclusion in the figure on saturation, and practically speaking, it presents some challenges, which precludes its inclusion in the figure in this manuscript. (Middle of page 13).*

Reviewer 2:

Chung and colleagues have revised their review of best practices for multi-species RNA-seq analysis. While it is improved, there are still a number of outstanding issues that need to be corrected. I would stress that my main concern is that this will be read by students and non-experts, and really must be accurate and clear so as not to propagate confusion.

Major issues:

Major issue 1:

The statement that "Genes with … high abundance should be excluded before downstream analyses" remains on line 292. After reading this section multiple times, my best guess at the authors' rationale for this is one or two highly variable, high abundance genes might make automatic color scale ranges in heatmaps so large that it is difficult to see variation in expression of other genes. This is not a terribly convincing reason, seeing as it can be easily solved by adjusting the color scale range as is commonly done.

As I stated in my review of the previous version, there may be particular analyses where this makes sense to do, but if so the authors need to explain this, clearly. To make this crystal clear, these are the problems with this statement and the framing in this section:

1. The positioning in the paper is such that it seems like the authors are implying high-abundance genes should be excluded before *all* downstream analyses, for instance differential expression analysis. There is *no* basis for that particular recommendation. It is possible to read this as only applying to cluster analyses, but even then it is not clear to me that this is really necessary in most cases besides for presentation purposes, and an article explicitly billing itself as setting out "best practices" should not contain this kind of ambiguity about such a basic issue.

2. The authors continue to confuse the reasons for experimental hemoglobin transcript depletion in reference 88. This depletion is to allow better quantification of the rest of the transcriptome, for the same reasons as one would do rRNA depletion, not because having a lot of hemoglobin transcript reads screws up downstream data analysis.

3. The authors cite four papers, including two of their own, to support this practice. I have gone through these papers and again find no support for this. I cannot pretend to know what exactly the authors are referring to with these papers, but I would note that according to their methods sections they do not filter high abundance genes before their own differential expression analysis. The do indeed not include 6S in their cluster analyses, but this is because they only perform cluster analysis on protein coding genes.

The closest I could find to an actual statement on the issue these papers were cited to support was in reference 41 where the authors write "this analysis of global gene regulation was conducted only on coding sequences (CDSs), precluding observation of differentially expressed noncoding RNAs, like the 6S RNA". Reference 70 also only analyzes coding genes as best as I can tell, and the only mention of 6S I could find is in a supplementary figure. The two references not by the authors of this review also do not seem to support this. Reference 89 makes no mention of 6S that I could find. Reference 90 does talk a bit about 6S/ssrS, but this seems to be because they have explicitly included it in their analyses and suggest it may be an important regulator for the process they are studying.

*We have re-worded in a way that we hope is suitable for everyone and included a highlight about TMM/Edge R, including an additional reference to “A scaling normalization method for differential expression analysis of RNA-seq data.” (middle of page 14) We discussed removing this, but this has been a persistent issue in analyses in my own group as well as reviews of manuscripts I handle, that I think it is important to include. While there are biological reasons this happens, and we point to one with the 6S RNA in Wolbachia, most of the time this relates to annotation issues. And as such, I suspect this may be more of an issue for researchers working with organisms that have less than stellar annotation. We are not saying that anyone must make these decisions, but everyone should be aware they may want to check. For some studies, researchers will need to assess this and make the appropriate decision for their study design.*

*For instance, I have a trainee where this just happened in the past two months because there are 4 mRNAs added that overlapped rRNA, which are likely bogus CDSs. One condition had far more rRNA contamination than the others, such that the corresponding 4 overlapping “mRNAs” dominated the counts in all the replicates for that condition. This is not just a matter of scaling, as reviewer 2 suggests, and we assure the reviewer that we know how to adjust the scales on heatmaps. Importantly, many analyses were affected but particularly clustering and similar types of analyses. The only solution was to remove those 4 mRNAs from the analysis and limit the analysis to CDSs. We suspect this is common for non-model organisms as it happens in our group frequently. We suspect that most of the time these cases lead to annotation improvements and as such are not reported in manuscripts and don’t really have supporting citations. However, it is something researchers should be aware of, particularly beginners working with non-model organisms. We use as an example our experience with the 6S RNA, which dominates the transcriptional profile at some life stages, sometimes being 75% of the mapped reads, and confounded clustering analyses, including WGCNA.*

*It is unclear if the reviewer objects to the bioinformatic removal of data. However, if one were to object to the bioinformatic removal of these transcripts, one would also need to object to the experimental removal of rRNA and hemoglobin. The fact that the experimental removal is acceptable for these analyses, indicates that the bioinformatic removal of similar transcripts should also be acceptable. In our hands, it improves the visualization and analysis of the data, in a manner that makes the data more approachable for biologists. I think our paper in MSystems balances this well, highlighting how it is both possible and important to do this type of filtering while still describing the transcriptional profile of the transcript removed. In this case, we removed the 6S RNA and described its transcription separately. After removing the 6S RNA, the Wolbachia PCA and WGCNA analyses look appropriate.*

Major Issue 2:

The authors continue to state that "treating every gene as an independent variable for bacterial samples leads to errors in variance estimations", on line 355. In their response to my previous comment on the issue, the authors state "these papers [that I cited] discuss independence in a different manner, that one gene going up has to be met with all others going down by the same amount given that relative abundance to one another is the approach." This is false. Lönnstedt and Speed 2002 and Smyth 2004 introduced the empirical Bayes method for estimating the variance in expression data, which is what the authors claim is being confounded by dependence between gene expression level measurements. If the authors would prefer an explicit reference for RNA-seq data I would direct them to Robinson and Smyth, Biostatistics 2008, which states: "We assume that all tags are independent for the purposes of estimation and inference. This is not strictly true since some sets of genes are involved in related biological functions and are inherently coregulated in their expression levels. However, our primary concern is getting an unbiased estimate of the dispersion parameter and consistency of the estimator is unaffected by this correlation."

I would like to reiterate here that I agree with the authors that the ultimate object of quantification should be transcripts, since this is what RNA-seq is measuring. What I object to is ascribing the problem with not doing this to some vague statistical issue that likely does not exist.

The authors now include a simulation study the authors claim shows that modeling coding sequences as independent variables leads to problems in variance estimation. While the details of this simulation study are inadequately described (e.g. what were the effect sizes? Are overlapping transcript models included? If so, how are they dealt with? How many reads were generated?), I see no reason to doubt the basic conclusion that one could more accurately detect differential expression with long transcripts than shorter genes. This does not imply anything about the variance estimation procedure, as this simulation conflates *at least* three separate issues: different accuracy in quantification against different gene models, differences in the amount of data for the different objects of quantification, and then possibly issues in the statistical model underlying the analysis.

The simplest explanation for these simulation results is that as differential expression tools work on count data, and longer transcripts generate more reads, it will simply be easier to detect smaller changes in expression for longer transcripts as there is more data. This is a well-known bias in RNA-seq data (see Young et al. "Gene ontology analysis for RNA-seq: accounting for selection bias" Genome Biology 2010). To show that this is a problem with variance estimation would require a much more involved simulation where the dispersion parameter is directly measured and manipulated, but to be honest this doesn't make a lot of sense to even attempt unless the authors are really committed to showing dependence relationships affect variance estimation.

*We have modified the text to show the observation of the difference between using CDS and transcript annotation, and we have left out any explanation for that difference. The simulated data is from a previous paper that we have cited, but we have added more details to the text so that the reader does not have to go look it up. (Middle of page 16).*

Minor issues:

From my previous comments: 2-17 Line 273: As far as I am aware TPMs were introduced in Li et al. Bioinformatics 2010 (ref 59), and this should be cited.

Author reply: We added a reference to TPM in Li et al. 2011 BMC Bioinformatics on line 325.

The correct reference is Li et al. "RNA-seq gene expression estimation with read mapping uncertainty" Bioinformatics 2010. Feel free to cite Li et al BMC Bioinformatics 2011 as well, though. Neither is currently cited on line 325 (ref 92 remains Wagner et al Theory Biosci 2013).

*This is updated to Li Bioinformatics 2010. (Middle page 15).*

Line 48: Reference 15 did not coin the term "dual-species transcriptomics", it coined the term "dual RNA-seq". The phrase "dual-species transcriptomics" is not used in the article.

*Sentence was rephrased and reworked to remove the implication that the reference referred to the coining of that phrase. The rephrasing was done so as to preserve this citation. (Middle page 3).*

Line 88: there is a disconnect between the statement in the paper that "techniques with longer processing time need to incorporate steps to minimize further changes in the transcriptome", and their insistence in their reply to reviewers that because only a single study has done this it is impossible to give any reasonable advice or leads for further research. Is it necessary or not? If it is necessary more than one study must have done this? As I previously wrote, surely there is some literature around this it is possible to extrapolate from, possibly in the single-cell or meta'omics literature? Westermann et al. PLoS Pathogens 2017 for instance includes a box on this issue, though is three years out of date now and was written before single-cell analysis really exploded.

*We say, “Techniques with longer processing time need to incorporate steps to minimize further changes in the transcriptome during sample preparation including RNA stabilization reagents. Selection of the right reagents depends upon the system and techniques used (e.g. as described in [13]),” where [13] is the Westermann paper from 2016 that I believe the reviewer requested last time. We have added the additional reference the reviewer requested. We believe this is sufficient. (Top of page 5).*

Line 173 and reply to reviewers: The authors seem to be under the impression that transcriptional complexity in bacteria is new. Studies looking at this transcriptome-wide date to at least 2009 (e.g. Güell et al., Science 2009; for one review see Wage and Grainger, "Pervasive transcription: illuminating the dark matter of bacterial transcriptomes" Nature Reviews Microbiology 2014, there are many others).

*We have added these references as well as a reference to a recently published manuscript that demonstrates direct RNA sequencing of bacterial RNA using ONT sequencing. (Bottom of page 8).*

Line 272: Do the authors mean exons?

*We rephrased this sentence. (Bottom of page 12).*

Line 286: what is "biological significance"?

*We removed this portion of the sentence. (Middle of page 13).*

Line 287: I still do not understand how one would account for batch effects in a fastq file, and certainly not how one would merge them after. To be clear, batch effects are generally assessed on the *count* level, which is after reads have already been mapped and quantified, and I'm not sure I even understand what it would mean to propagate this back to the fastq files and merge them after.

*We removed this portion of the sentence. (Middle of page 13).*

Line 289: The authors continue to say in regard to unsaturated libraries they should be discarded or "the analysis should be adjusted accordingly" and cite one of their own papers with no further explanation. This paper does seem to be possibly relevant (it just looks at highly expressed genes), but it is not obvious and took a fairly close reading to understand why this paper was being cited (the final section of the results discusses this a bit). Surely a review of best practices should provide a little more concrete advice.

*We re-phrased to give more options and to provide more details for an alternate method of analysis, but leaving it open-ended so as not to imply these are the only ways to address this. (Middle of page 13).*

Line 289: prevented -> presented

*This has been corrected. (Middle of page 13).*

Line 350: if the authors are going to bring up sleuth and bootstrap estimates, it would be good to specify what this is for, as they are used to estimate technical variation in quantification and not for count normalization as the placement in this paragraph implies. If so, it may also be worth including the swish paper here (Zhu et al NAR 2019).

*We have added a reference to Swish and reworked the sentences to accommodate its inclusion. . (Middle of page 16).*

References: three references I mentioned in my previous review (30-32) are in the reference list, along with reference 33, and never cited. I don't particularly care if they're cited or not, but this seems to be a formatting error.

*We apologize. References in Endnote persist while track changes is on, and that was the version we thought we would need to submit. However, we found we needed to submit a copy without track changes and did not rerun the citation manager between accepting changes and making the version the reviewer used. That has been fixed.*
